# Whole-genome Sequence Analysis Revealed Novel Subjective Cognitive Decline-associated Genes in 10,763 Chinese

**DOI:** 10.1093/gpbjnl/qzaf063

**Published:** 2025-07-29

**Authors:** Mengying Wang, Liyang Sun, Xin Xu, Ruoqi Dai, Qilong Tan, Yun Zhu, Andi Xu, Weifang Zheng, Yuanxing Tu, Dan Zhou, Wenyuan Li, Xifeng Wu

**Affiliations:** Center of Clinical Big Data and Analytics of the Second Affiliated Hospital and School of Public Health, Zhejiang University School of Medicine, Hangzhou 310058, China; Department of Nutrition, Child, and Adolescent Health, School of Public Health, Hangzhou Medical College, Hangzhou 310007, China; The Second Affiliated Hospital of Zhejiang University School of Medicine, Lanxi Branch (Lanxi People’s Hospital), Lanxi 321100, China; Center of Clinical Big Data and Analytics of the Second Affiliated Hospital and School of Public Health, Zhejiang University School of Medicine, Hangzhou 310058, China; Center of Clinical Big Data and Analytics of the Second Affiliated Hospital and School of Public Health, Zhejiang University School of Medicine, Hangzhou 310058, China; Center of Clinical Big Data and Analytics of the Second Affiliated Hospital and School of Public Health, Zhejiang University School of Medicine, Hangzhou 310058, China; Center of Clinical Big Data and Analytics of the Second Affiliated Hospital and School of Public Health, Zhejiang University School of Medicine, Hangzhou 310058, China; Center of Clinical Big Data and Analytics of the Second Affiliated Hospital and School of Public Health, Zhejiang University School of Medicine, Hangzhou 310058, China; Lanxi Hospital of Traditional Chinese Medicine, Lanxi 321100, China; The Second Affiliated Hospital of Zhejiang University School of Medicine, Lanxi Branch (Lanxi People’s Hospital), Lanxi 321100, China; Center of Clinical Big Data and Analytics of the Second Affiliated Hospital and School of Public Health, Zhejiang University School of Medicine, Hangzhou 310058, China; Center of Clinical Big Data and Analytics of the Second Affiliated Hospital and School of Public Health, Zhejiang University School of Medicine, Hangzhou 310058, China; Center of Clinical Big Data and Analytics of the Second Affiliated Hospital and School of Public Health, Zhejiang University School of Medicine, Hangzhou 310058, China; Zhejiang Key Laboratory of Intelligent Preventive Medicine, Hangzhou 310058, China; National Institute for Data Science in Health and Medicine, Zhejiang University, Hangzhou 310058, China

**Keywords:** Whole-genome sequencing, Subjective cognitive decline, Genetic risk factor, Cognitive decline, Rare variant

## Abstract

Subjective cognitive decline (SCD) is widely regarded as a potential preclinical stage of Alzheimer’s disease (AD), yet its genetic basis remains poorly understood. To address this gap, we investigated genetic biomarkers associated with SCD using whole-genome sequencing (WGS) in 10,763 Chinese participants from the Healthy Zhejiang One Million People Cohort (HOPE Cohort). The discovery stage included 9284 samples, with 1479 samples used for validation. Using a two-stage design, we systematically investigated both common and rare variants associated with SCD. In rare variant analyses, we identified and replicated an association between the upstream region of *SEPHS2* and SCD. *SEPHS2* is involved in selenophosphate synthesis, and a Mendelian randomization analysis reveals that its expression levels in both blood and brain cerebellum are associated with AD. Additionally, we identified *CLVS2*, which encodes a protein primarily expressed in neuronal cells, as a potential regulator for SCD based on missense rare variants. Multi-omics evidence suggests that both *SEPHS2* and *CLVS2* may play roles in neurodegenerative diseases. For common variants, we validated 8 known loci related to cognitive decline, 3 of which originated from the only existing SCD genetic study conducted under a migraine background. Overall, our WGS-based study fills the gap in SCD research by providing vital genetic evidence from an East Asian population and offers insights into the pathogenic mechanisms of SCD.

## Graphical abstract

**Figure:**
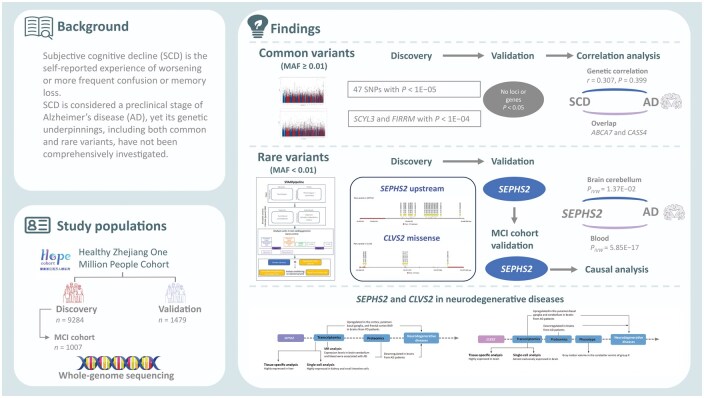

## Introduction

Dementia is a syndrome of deterioration in cognitive function that mainly affects elderly people, and its global prevalence is projected to soar from the current 55 million to 78 million by 2030 [1,2] (https://www.who.int/news-room/fact-sheets/detail/dementia). One area of the research has been focused on identifying novel biomarkers for the early clinical stage of dementia, known as subjective cognitive decline (SCD) [3–5]. SCD is the self-reported experience of worsening or more frequent confusion or memory loss [6]. It refers to a form of cognitive impairment that is observed in the early phases of dementia. SCD has a prevalence between 6.1% and 52.7% and is generally higher in Asians than in Caucasians [7,8]. According to the records on the NHGRI-EBI Catalog of human genome-wide association studies (GWAS Catalog) website, 455 single nucleotide polymorphisms (SNPs) were reportedly potentially associated with cognitive decline measurements from studies, and most of the studies were conducted primarily in European populations (https://www.ebi.ac.uk/gwas/efotraits/EFO_0007710). A study conducted within the East Asian population revealed an association between several common genetic variants and SCD in individuals with migraines (*n* = 1019) [9]. The genetic results might be affected by migraine genetics because the study wasn’t done in a broader population and had a small sample size. To deepen our understanding of the genetic etiology of SCD, it is imperative to conduct studies involving larger and more representative samples.

Few studies have employed whole-genome sequencing (WGS) in investigating SCD in the general population, whether in East Asia or other ethnic groups. Some research has looked into the connection between genes related to Alzheimer’s disease (AD) and cognitive decline with SCD. Previous research has linked SCD to the well-known apolipoprotein E (*APOE*) gene, a genetic risk factor for late-onset AD [10,11]. Meta-analyses with sample sizes ranging from 5000 to 10,000 in mixed ethnicities showed marginal associations between *APOE ε4* and SCD [12]. In older individuals, significant associations between *APOE ε4* and memory decline as well as global cognitive function were also reported (*n* = 3045) [13]. Additionally, a meta-analysis of genome-wide association studies (GWAS) conducted using five UK cohorts of older adults (*n* = 3511) demonstrated a significant association between *TOMM40* and *APOE* with age-related cognitive decline, and the results were further replicated in three independent Swedish cohorts (*n* = 1367) [14]. Moreover, SNPs in various genes, including *ADAMTS9*, *BDNF*, *CASS4*, *COMT*, *CR1*, and *DNMT3A*, were found susceptible to age-related cognitive decline [15]. Although SCD has been identified as a predictor of cognitive decline [16] and dementia, establishing a genetic relationship among them remains challenging.

While some studies have conducted rare variant analyses on cognitive function and AD, similar analyses on SCD have not been reported in the Chinese population. GWAS have identified biological pathways of potential relevance to cognitive function [17]. However, the investigation of rare variants of SCD, which may have larger phenotypic effects, has been relatively limited. A recent exome study conducted in the UK Biobank (*n* =  485,930) examined rare protein-coding variants impacting adult cognitive function (educational attainment, reaction time, and verbal-numerical reasoning) and found that eight genes (*ADGRB2*, *KDM5B*, *GIGYF1*, *ANKRD12*, *SLC8A1*, *RC3H2*, *CACNA1A*, and *BCAS3*) were significantly associated [18]. Additionally, in the known AD-related genes, 68 coding rare variants were reported in the amyloid precursor protein (*APP*) gene, 321 in the presenilin 1 (*PSEN1*) gene, and 63 in the presenilin 2 (*PSEN2*) gene (https://www.alzforum.org/mutations). While rare variants demand exceptionally large sample sizes for detection, they possess greater potential for significant phenotypic impacts compared to common variants. Therefore, it is imperative to investigate the underlying etiology of SCD and elucidate the role of these rare variants in its manifestation.

Therefore, we conducted WGS on 9284 discovery and 1479 validation samples from individuals with SCD measurements, using data from the Healthy Zhejiang One Million People Cohort (HOPE Cohort) in China. Our comprehensive analyses included functionally informed WGS association analyses of both common and rare variants, incorporating single-variant, gene-level, gene-centric, and non-gene-centric approaches. We also validated known loci associated with cognitive decline within our dataset and investigated the genetic association between SCD and AD. Additionally, we examined the novel genes identified using published multi-omics databases and employed Mendelian randomization (MR) analysis to explore their causal role in AD, providing insights into the pathophysiological mechanisms underlying SCD and neurodegenerative diseases.

## Results

### Overview

We analyzed WGS data from 10,763 Chinese individuals in the HOPE Cohort to identify SCD-related variants and genes. The discovery cohort consisted of 9284 individuals, while the validation cohort included 1479 participants. Their mean ages were 58.8 [standard deviation (SD) = 9.8] and 62.5 (SD = 7.6) years, respectively, and the female-to-male ratios were 1.6:1 and 1.2:1. To emphasize, the validation cohort was a separate cohort from the discovery cohort. All analyses were initially performed in the discovery cohort and then validated in replication samples. The Ascertain dementia 8 (AD8) score was implied as the response variable for both common and rare analyses.

**Figure 1:**
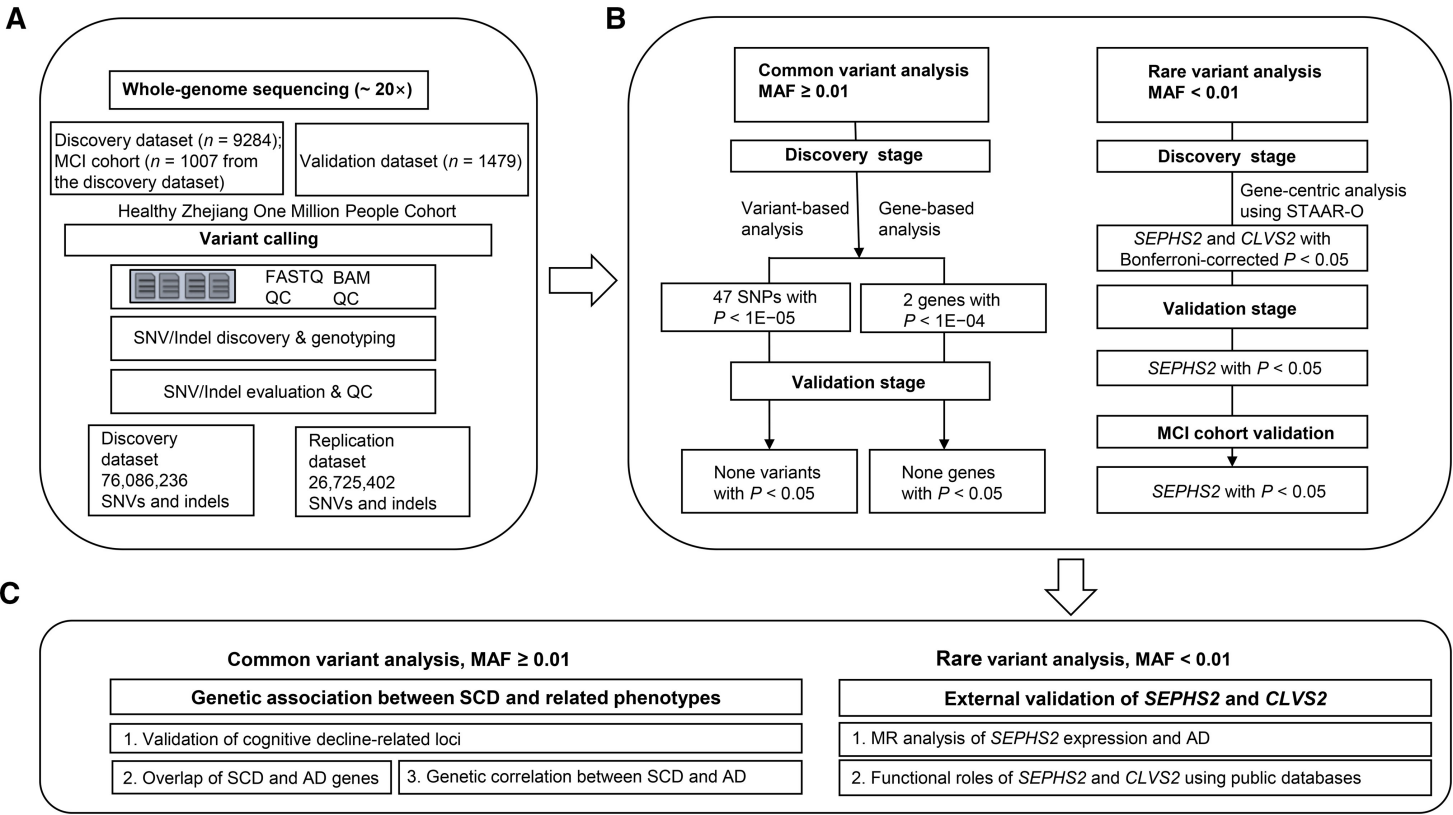
Study workflow **A**. Whole-genome sequencing design. Variants that passed QC were extracted from discovery and validation datasets, respectively. **B**. Variant association analysis. Variants that met MAF ≥ 0.01 and *P* < 1E−05 in the discovery dataset were tested in the validation dataset. Variants with MAF < 0.01 were aggregated as rare variants and then underwent gene-centric analysis. Variants with Bonferroni-corrected *P* < 0.05 in the discovery stage were then sent to be replicated. **C**. Association of SCD with related phenotypes and external validation of *SEPHS2* and *CLVS2*. QC, quality control; SNV, single nucleotide variant; MAF, minor allele frequency; STAAR-O, omnibus test in the STAARpipeline; MR, Mendelian randomization; AD, Alzheimer’s disease; STAARpipeline, the variant-Set Test for Association using Annotation infoRmation pipeline; SNP, single nucleotide polymorphism; MCI, mild cognitive impairment; indel, insertion and deletion; FASTQ, a text-based format for storing biological sequence reads and their corresponding quality scores; BAM, binary alignment/map; SCD, subjective cognitive decline.

First, we introduced the sample characteristics and results of WGS. Second, we conducted GWAS with age, sex, and 20 principal components as control variables. For the consequent analysis, we compared reported variants and genes associated with cognitive decline to our dataset, and conducted linkage disequilibrium score regression (LDSC) with public AD GWAS summary data to explore its genetic associations with SCD. Third, we conducted a gene-centric analysis focused on rare variants [minor allele frequency (MAF) < 0.01] to identify genomic regions associated with SCD, using the same covariates as in the GWAS analysis. We performed an MR analysis to identify associations between the expression of identified genes and AD and further validated these findings using external public multi-omics databases. **Figure 1** outlines the study workflow.

**Figure 2:**
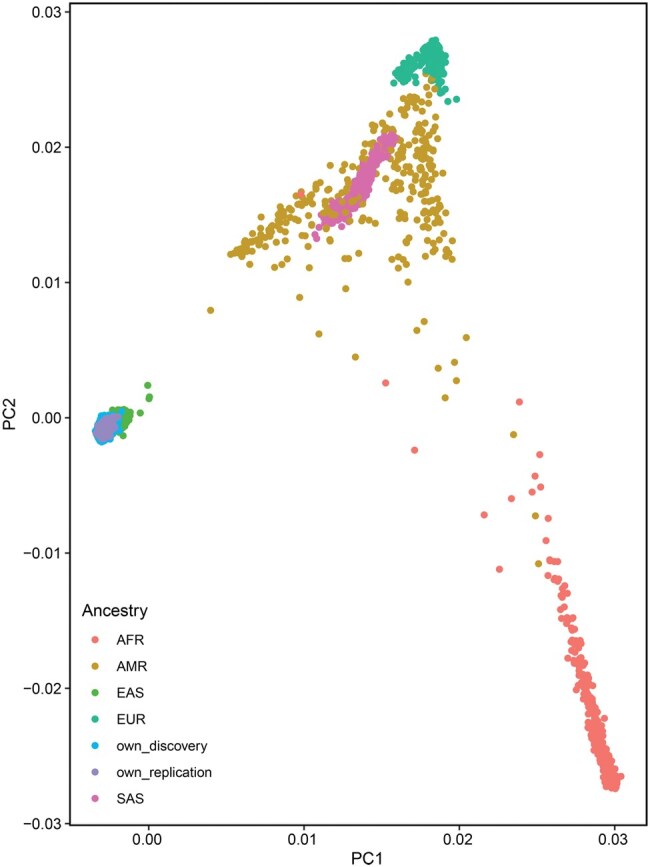
PCA of participant genetic background The first two PCs show that both the discovery and replication cohorts from our study (own_discovery and own_replication) highly overlapped and belonged to EAS population. PCA, principal component analysis; PC, principal component; AFR, African; AMR, Admixed American; EAS, East Asian; EUR, European; SAS, South Asian.

A total of 81.4 million and 28.6 million autosome biallelic variants were obtained from WGS for discovery and replication samples, respectively. Variant calling was performed using Genome Analysis Toolkit (GATK). A total of 76,086,236 (discovery) and 26,725,402 (validation) genetic variants [single nucleotide variants (SNVs) and short insertions and deletions (indels)] from discovery and replication samples passed stringent quality control (QC) criteria for both genotypes and samples (see Materials and methods). And 46,844,086 (discovery) and 21,256,496 (validation) variants were found in the single nucleotide polymorphism database, build 154 (dbSNP154). In the discovery dataset, the majority of SNVs were located in intronic (277,786,050) downstream (35,781,318), and upstream (34,112,817) regions. Exonic variants totaled 4,912,810, comprising 1,253,515 synonymous and 2,169,011 missense mutations. Additionally, we identified 3,767,089 and 3,015,622 variants in untranslated regions (UTRs) and transcription factor (TF) binding sites, respectively. A total of 889,196 variants were classified as deleterious by Sorting Intolerant From Tolerant (SIFT) (score < 0.05), while Polymorphism Phenotyping (PolyPhen) categorized 582,076 variants as probably damaging (score > 0.908) and 371,402 as possibly damaging (0.446 < score ≤ 0.908), respectively. Principal component analysis (PCA) confirmed the East Asian genetic ancestry of the participants, as depicted in **Figure 2**.

### Results of common variant analysis

Common variants (MAF ≥ 0.01) were included in the single-variant analysis. The Manhattan plot revealed prominent peaks on chromosomes 4 and 22 (Figure S1A and B), with 47 loci surpassing the suggestive genome-wide significance threshold (*P* < 1E−05) and the most significant locus having the lowest *P* value of 7.13E−07. Among these loci, rs254559, an ncRNA_intronic variant mapped to *C5orf66*, was replicated in the validation samples (*P* = 0.04), albeit with an opposite effect size (Table S1). The genomic inflation factors (λ) for discovery and validation analyses were 1.007 and 0.994, respectively, indicating no inflation in the test statistics (Figure S1C and D).

In the gene-based analysis, we identified the top 20 significant genes in the discovery stage, with *SCYL3* (*P* = 3.91E−05) and *FIRRM* (*P* = 6.12E−05) (Table S2). *FIRRM* showed a trend toward significance in the replication stage (*P* = 0.052). Gene set enrichment analysis (GSEA) highlighted key biological processes, with the most significant gene sets pertaining to immune response inhibition, diacylglycerol (DAG) and inositol 1,4,5-trisphosphate (IP3) signaling, central nervous system (CNS) myelin maintenance, pons-specific expression, and Ca^2+^/calmodulin-dependent protein kinase (CaMK IV)-mediated phosphorylation of cAMP response element-binding protein (CREB) (Table S3).

We further performed external validation using publicly available data. First, by querying the GWAS Catalog, we identified 455 variants that were associated with cognitive decline (Table S4). Of these, 8 SNPs (rs13121626, rs13243882, rs17111203, rs2473238, rs76600213, rs144191744, rs4488224, and rs7985509) were also statistically significant (*P* < 0.05) in our discovery or replication dataset (**Table 1**). Second, based on a previously reported meta-analysis of 7 European ancestry datasets [19], we found that 12 out of 17 reported AD-related genes exhibited *P* < 0.5 in our discovery dataset. Particularly, *ABCA7* and *CASS4* showed *P* values of 0.018 and 0.049, suggesting a potential shared genetic risk between SCD and AD (Table S5). Third, we conducted a genetic correlation analysis between SCD (our data) and AD (a Japanese cohort), and the estimated genetic correlation was 0.307 (*P* = 0.399), possibly due to the small sample size.

**Table 1:** Loci significantly associated with cognitive decline from GWAS Catalog in the HOPE Cohort

Chr	Pos (bp)	SNP ID	Mapped gene	GWAS Catalog					HOPE Cohort					*P*
				A1	Beta	OR	CI	*P*	A1	N	AF	Beta	SE	
4	10,489,927	rs13121626_a_	*CLNK*	A	0.51	/	[0.30–0.72]	2.00E−06	C	9284	0.64	−0.06	0.03	0.031
7	28,247,668	rs13243882_b_	*JAZF1–AS1*, *PPIAP80*	T	0.18	/	/	6.00E−06	A	9284	0.83	0.08	0.04	0.027
1	94,180,440	rs17111203_c_	*ARHGAP29*	/	/	0.17	[0.08–0.36]	6.00E−07	G	9284	0.09	0.11	0.05	0.022
1	22,263,838	rs2473238_d_	*WNT4*, *MIR4418*	G	0.17	/	/	9.00E−06	C	9284	0.08	0.10	0.05	0.047
1	60,526,484	rs76600213_a_	*LINC01748*	T	0.99	/	[0.56–1.43]	8.00E−06	A	9284	0.04	0.15	0.07	0.039
1	91,730,849	rs144191744_e_	*TGFBR3*	/	/	0.12	[0.05–0.29]	3.00E−08	C	1479	0.02	−0.58	0.25	0.019
11	87,510,788	rs4488224_c_	*PSMA2P1*, *RNU6–1135P*	/	/	0.23	[0.13–0.40]	1.00E−07	A	1479	0.44	0.20	0.07	0.002
13	81,170,245	rs7985509_a_	*DPPA3P3*, *LINC00564*	G	0.82	/	[0.54–1.10]	1.00E−08	C	1479	0.45	−0.14	0.07	0.040

*Note*: The subscripts of SNP IDs denote trait names: a, rate of cognitive decline in AD; b, age-related cognitive decline (memory); c, subjective cognitive decline in chronic migraine; d, age-related cognitive decline (language); e, subjective cognitive decline in migraine, in episodic migraine, and migraine without aura. HOPE Cohort, Healthy Zhejiang One Million People Cohort; Chr, chromosome; Pos, position; OR, odds ratio; CI, confidence interval; N, sample size; AF, allele frequency; SE, standard error; SNP, single nucleotide polymorphism; GWAS, genome-wide association study; AD, Alzheimer’s disease.

### Results of rare variant analysis

**Figure 3:**
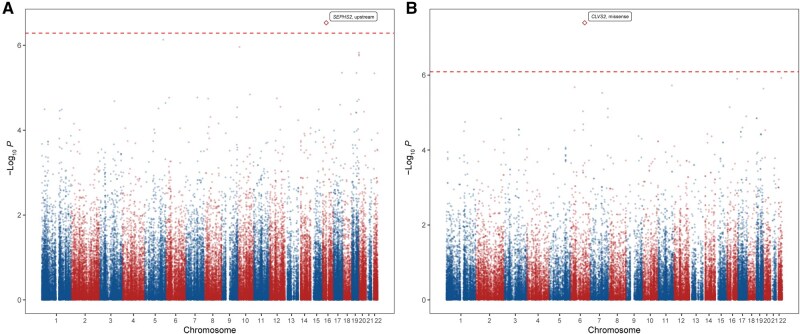
Manhattan plots of rare variant set analysis **A**. Manhattan plot of the rare coding variant set in the discovery stage. Dots in alternating blue and red represent functional category-based coding genes located on odd- and even-numbered chromosomes, respectively. The red dashed line indicates the Bonferroni-corrected significance threshold (*P* < 5E−07) for coding categories. **B**. Manhattan plot of the rare non-coding variant set in the discovery stage. Dots in alternating blue and red represent functional category-based non-coding genes located on odd- and even-numbered chromosomes, respectively. The red dashed line indicates the Bonferroni-corrected significance threshold (*P* < 3.57E−07) for non-coding categories.

**Figure 4:**
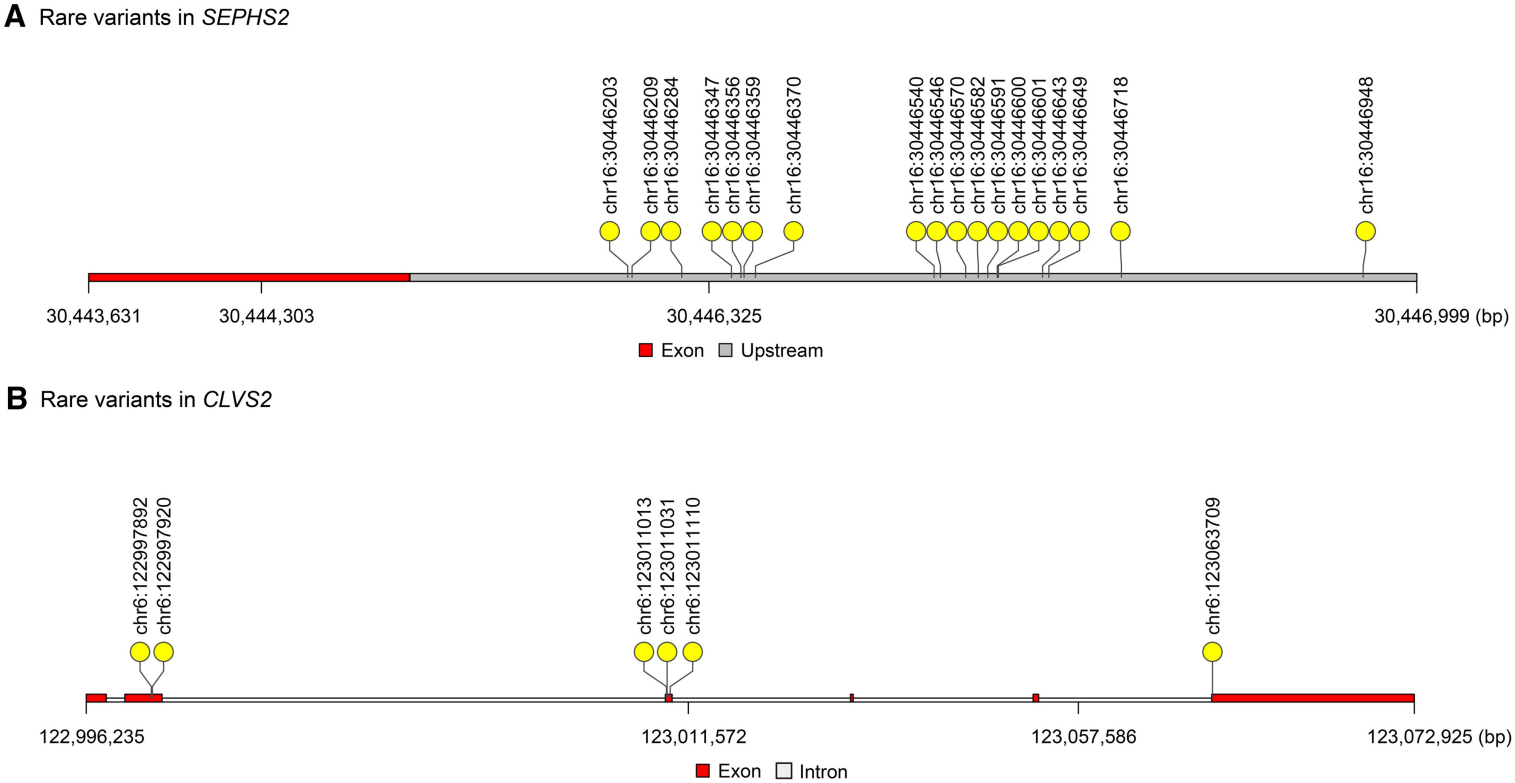
Distribution of rare variants in *SEPHS2* and *CLVS2* **A**. Lollipop plot showing the distribution of rare variants in the upstream region of *SEPHS2*. **B**. Lollipop plot showing the distribution of rare variants in the exonic regions of *CLVS2*.

We grouped rare coding and non-coding variants (MAF < 0.01) based on the functional categories of their corresponding genes. **Figure 3** presents the Manhattan plots of the rare coding and non-coding variant set analyses, while Figure S2 provides the Manhattan plots of the protein-truncating variant (PTV) and PTV plus disruptive missense (PTV + D) results. Two variant sets were identified to be significantly associated with SCD: one in the upstream region of *SEPHS2* (*P* = 2.95E−07) and the other in the missense aggregate set situated within the coding region of the clavesin-2 (*CLVS2*) gene (*P* = 4.02E−08). In the validation assessments, we successfully replicated the upstream category in *SEPHS2* (*P* = 0.026), but were unable to aggregate the missense set in *CLVS2*. It was possibly due to the shorter exons of *CLVS2*, where there were not enough rare variants falling within its exonic regions to reach statistical significance in the replication stage (**Table 2**). **Figure 4** shows the specific location of each variant in the non-coding region of *SEPHS2* and the coding region of *CLVS2*. The characteristics of rare variants accounting for *CLVS2* missense are listed in Table S6.

**Table 2:** Rare variant sets in coding regions of *CLVS2* and non-coding regions of *SEPHS2*

Gene name	Category	Discovery					Validation				
		nSNV	SKAT	Burden	ACAT-V	STAAR-O	nSNV	SKAT	Burden	ACAT-V	STAAR-O
*SEPHS2*	Upstream	18	1.58E−03	3.86E−04	9.50E−08	2.95E−07	3	0.012	0.077	0.081	0.026
	Downstream	22	0.367	0.482	0.570	0.468	8	0.564	0.214	0.215	0.358
	Enhancer_CAGE	28	0.680	0.614	0.561	0.633	35	0.881	0.623	0.623	0.788
	Enhancer_DHS	57	0.589	0.937	0.359	0.741	57	0.498	0.678	0.676	0.607
	Promoter_CAGE	28	0.680	0.614	0.561	0.633	35	0.881	0.623	0.623	0.788
	Promoter_DHS	65	0.752	0.913	0.531	0.865	64	0.499	0.705	0.703	0.626
	UTR	28	0.838	0.272	0.754	0.676	10	0.789	0.393	0.394	0.574
*CLVS2*	Missense	6	1.63E−08	1.03E−03	1.05E−03	4.02E−08	/	/	/	/	/
	Ssynonymous	8	0.604	0.698	0.591	0.639	2	0.534	0.542	0.535	0.535

*Note*: Rare variant set analysis was performed using STAARpipeline, with the STAAR-O *P* value as the criterion for association. nSNV, the number of SNVs aggregated in a specific category region of the gene; SNV, single nucleotide variant; SKAT, sequence kernel association test; ACAT-V, aggregated Cauchy association test combining variant-level *P* values; STAAR-O, omnibus test in the STAARpipeline; STAARpipeline, the variant-Set Test for Association using Annotation infoRmation pipeline; CAGE, cap analysis of gene expression; DHS, DNase I hypersensitive site; UTR, untranslated region.

In an internal validation set including 706 mild cognitive impairment (MCI) cases and 301 controls, enhancer_CAGE, enhancer_DHS, and promoter_CAGE in the non-coding region of *SEPHS2* were found to be associated with MCI (all *P* < 0.05) (Table S7). Furthermore, conditional analysis incorporating 22 loci associated with cognitive decline from the GWAS Catalog (selected from 455 before pruning) showed that both *SEPHS2* and *CLVS2* retained their original *P* values (Table S8), indicating the robustness of our findings.

Using a significance threshold of *P* < 2.50E−06, the missense variant of *PCED1A* was statistically significant in both the discovery dataset (*P* = 2.30E−06) and the validation dataset (*P* = 0.032), as well as its promoter_CAGE, enhancer_DHS, and UTR regions. The coding regions of *MPZL3*, *OX4I1*, *CRYM*, *SAMM50*, and *CCDC167*, along with the non-coding regions of *ADRA1B* and *FAM107B*, were found to be associated with SCD only in the discovery dataset (Table S9).

We then conducted an MR analysis to assess causality with AD using data of European ancestry. The instrumental variables (IVs) are listed in Table S10. We found that *SEPHS2* expression in both blood and cerebellum was causally associated with AD, with inverse variance weighted *P* values of 5.85E−17 and 1.37E−02, respectively (**Table 3**), but no association was observed in the brain cerebellar hemisphere. No significant heterogeneity or pleiotropy was detected in the analysis (all *P* > 0.05). However, MR analysis for *CLVS2* could not be conducted due to an insufficient number of IVs.

**Table 3:** MR analysis of *SEPHS2* expression and AD

Sample	Method	nSNP	Beta	SE	*P*
Blood	MR-Egger	180	5.27E−04	2.94E−04	7.49E−02
	Weighted median	180	8.59E−04	1.39E−04	6.57E−10
	Inverse variance weighted	180	8.12E−04	9.71E−05	5.85E−17
	Simple mode	180	9.53E−04	3.08E−04	2.32E−03
	Weighted mode	180	9.01E−04	2.52E−04	4.53E−04
Brain — Cerebellum	MR-Egger	19	4.20E−04	8.27E−04	6.18E−01
	Weighted median	19	5.08E−04	2.13E−04	1.73E−02
	Inverse variance weighted	19	4.19E−04	1.70E−04	1.37E−02
	Simple mode	19	5.73E−04	3.53E−04	1.22E−01
	Weighted mode	19	5.69E−04	3.61E−04	1.33E−01
Brain — Cerebellar hemisphere	MR-Egger	9	−7.01E−04	2.26E−03	7.66E−01
	Weighted median	9	6.58E−05	2.22E−04	7.67E−01
	Inverse variance weighted	9	1.07E−04	1.84E−04	5.63E−01
	Simple mode	9	5.03E−05	3.37E−04	8.85E−01
	Weighted mode	9	4.87E−05	3.11E−04	8.80E−01

*Note*: The *cis*-eQTL dataset from blood samples was downloaded from the eQTLGen consortium dataset, and the dataset from brain cerebellum and cerebellar hemisphere samples was downloaded from the GTEx Portal, extracting genetic variants significantly associated with the expression of *SEPHS2*. Outcome data of AD (ieu-b-5067), with 954 AD cases and 487,331 controls of European ancestry, were from the IEU OpenGWAS project. MR, Mendelian randomization; *cis*-eQTL, *cis*-expression quantitative trait locus; GTEx, Genotype-Tissue Expression Project.

### Supporting evidence from a multi-omics perspective in public datasets

**Figure 5:**
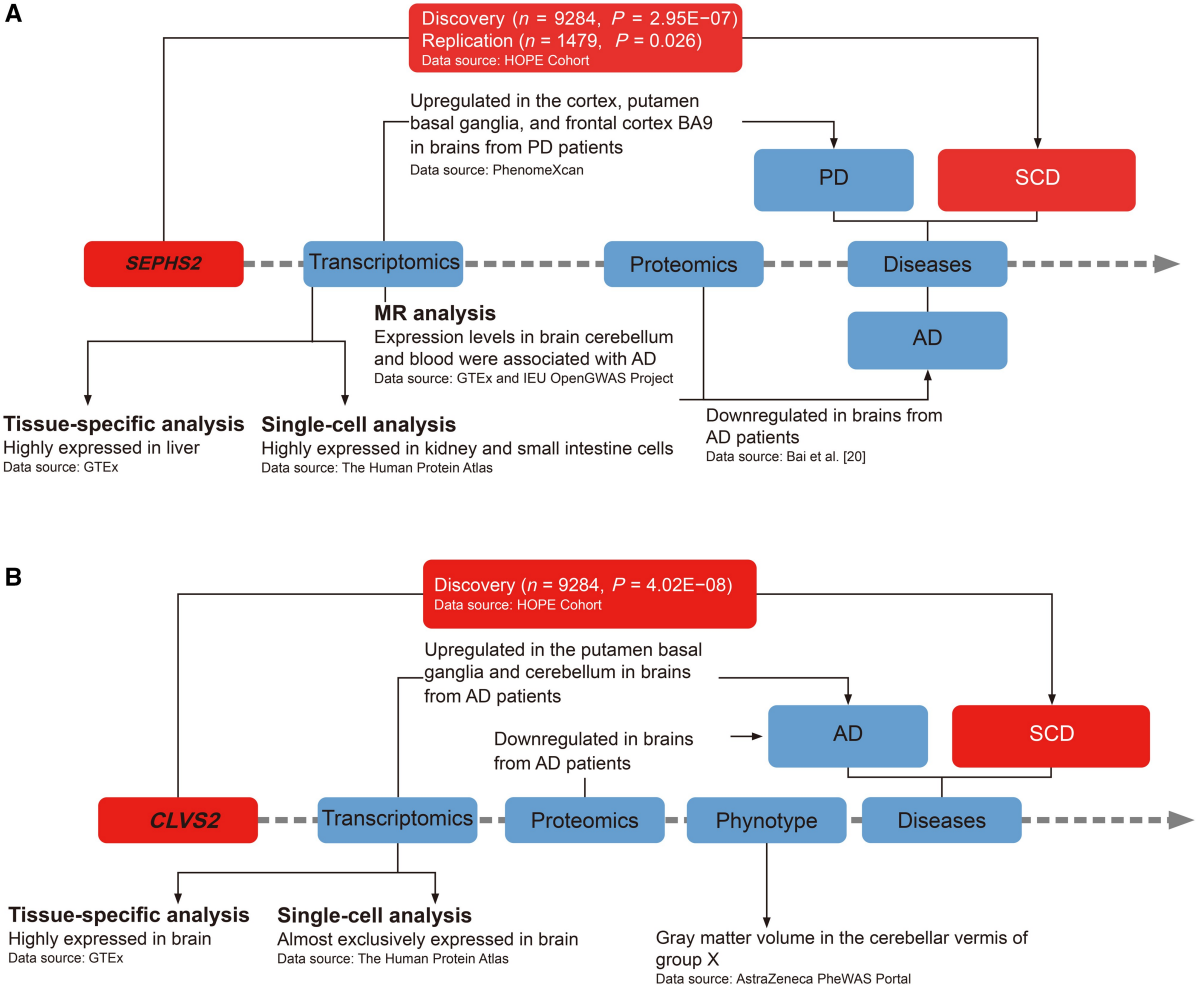
Multi-omics characterization of *SEPHS2* and *CLVS2* **A**. Integrated multi-omics data for *SEPHS2*. **B**. Integrated multi-omics data for *CLVS2*. HOPE Cohort, Healthy Zhejiang One Million People Cohort; PD, Parkinson’s diseases; GTEx, Genotype-Tissue Expression Project.

We investigated the functional roles of *SEPHS2* and *CLVS2* using public multi-omics datasets, including transcriptome-, proteome-, and phenome-wide association studies. Overall, the available evidence for the roles of *SEPHS2* in AD or other neurodegenerative conditions was limited, but *CLVS2* exhibited strong associations with brain structure and neuronal function (**Figure 5**).

A meta-analysis of seven proteomics datasets focusing on brain tissues of AD patients revealed that both *SEPHS2* and *CLVS2* were downregulated (*P* = 6.92E−02 and *P* = 8.75E−03, respectively; Table S11) [20]. In the PhenomeXcan database [21], elevated transcription levels of *SEPHS2* were observed in brain tissues from patients with Parkinson’s disease (PD) (*P* = 0.005; Table S12). Based on RNA sequencing (RNA-seq) data from the Genotype-Tissue Expression Project (GTEx) v8, elevated expression of *CLVS2* was predominantly observed in multiple brain regions, while *SEPHS2* was predominantly expressed in the liver (https://www.gtexportal.org/home/) [22] (Figure S3). Notably, high expression of *CLVS2* was detected in the cerebellar hemisphere and cerebellum (Figure S3). Single-cell RNA-seq data further demonstrated that expression of the CLVS gene family was almost exclusively restricted to neuronal cells, including both inhibitory and excitatory neurons, as well as glial cells, while *SEPHS2* was highly expressed in epithelial cells (Figure S4). Additionally, lower expression of *CLVS2* in the cerebellum was associated with higher AD risk (*P* = 0.019) [21] (http://apps.hakyimlab.org/phenomexcan) (Table S12). In analyses using the UK Biobank 450k data, *CLVS2* was most significantly associated with the gray matter volume in the cerebellar vermis among continuous traits analyzed (*P* = 2.38E−05) (https://azphewas.com/) [23] (Table S13).

## Discussion

This study is the first to report SCD-associated GWAS results in a general population, identifying two novel genes, *SEPHS2* and *CLVS2*, through rare variant analysis. Furthermore, *SEPHS2* was found to be associated with AD in individuals of European ancestry based on the results of MR analysis. Our analysis revealed novel rare variants related to SCD and validated common variants linked to cognitive decline, broadening our understanding of the genetic underpinnings of cognitive decline.

In this study, we successfully applied the variant-Set Test for Association using Annotation infoRmation pipeline (STAARpipeline) [24–26] and discovered two novel SCD-related genes. The challenge of detecting rare variant signals has persisted, primarily due to the limitations imposed by small sample sizes capable of undergoing WGS, as well as difficulties in interpreting the functional implications of these rare variants. STAARpipeline provides a convenient association analysis for non-coding variants in WGS data, including integration of genotype data and their functional annotations, association analysis, result summary, and visualization, enables flexible and computationally efficient rare variant analysis, and has been used in many high-quality studies [27–29].

Recent studies focusing on AD and other neurodegenerative conditions have ventured into rare variant discoveries. For instance, a robust genetic association study encompassing 544,384 European and admixed European participants identified two rare missense variants — *APOE ε3* (V236E) and *APOE ε4* (R251G) — that were found to reduce the risk of AD substantially [30]. Additionally, a review presented several replicated genes that had been identified to harbor AD-associated rare variants (MAF < 1%). Those genes were *APP* [31], *PSEN1* [31], *PSEN2* [31], *SORL1* [32], *ABCA7* [33], *TREM2* [34], and others [35]. However, no studies have reported the rare variant analysis on SCD.

Our analysis highlighted two novel SCD-related genes, *SEPHS2* and *CLVS2*. We successfully replicated the association of *SEPHS2* in both the validation dataset and our MCI cohort, establishing it as a robust gene linked to cognitive decline. In eukaryotic cells, *SEPHS2* is responsible for the synthesis of selenophosphate, whereas *SEPHS1* is implicated in the maintenance of cellular redox homeostasis [36]. The enzyme selenophosphate synthase-2, encoded by *SEPHS2*, is directly involved in the biosynthesis of selenocysteine (Sec) and subsequent selenoproteins [37]. Previously, selenoproteins have been shown to play a pivotal role in inflammation-associated carcinogenesis via immune response modulation and oxidative stress regulation [38]. However, emerging research has begun to uncover the relationship between selenium, selenoproteins, and various neurodegenerative diseases [39]. Specifically, selenoproteins have been observed to colocalize with the pathological features of neurological disorders, such as neurofibrillary tangles and Lewy bodies [40,41]. Rare genetic variants localized upstream of the *SEPHS2* gene might influence selenoprotein levels, though this possibility has not yet been explored. It was reported that selenoprotein deficiencies were likely to promote the accumulation of reactive oxygen species (ROS) [39,42], leading to mitochondrial damage, neuroinflammation, and ultimately neuronal death. As we observed in our study, the elevated expression levels of *SEPHS2* were observed in individuals with PD, hinting that *SEPHS2* might function to counterbalance neurodegeneration.

*CLVS2* is a paralog of clavesin-1 (*CLVS1*), and collectively, they encode the clavesin protein family. These proteins are predominantly localized on clathrin-coated vesicles (CCVs) and serve as critical regulators for the morphology of endosomes and lysosomes [43]. A missense variant in the *CLVS2* gene results in a decrease in clavesin protein levels within neurons, which may impair the functions of CCVs and lysosomes, particularly in pathways related to CCV-mediated transport. Given that lysosomes play a pivotal role in both the degradation of macromolecules and the induction of apoptotic cell death [44], any dysfunction in these organelles could increase neuronal susceptibility to apoptosis, thereby accelerating neuronal death [43,45]. Moreover, a proteomic study focusing on brain cortical specimens demonstrated significantly reduced levels of clavesin-2 and clavesin-1 proteins in AD patients compared to controls (*P* = 8.75E−03 and *P* = 8.64E−03, respectively) [20]. Notably, *CLVS2* expression was predominantly found in neuronal tissues, specifically in the cerebellar hemisphere and cerebellum. The cerebellum, whose primary function is to regulate balance, has also been implicated in neurodegenerative diseases like AD and amyotrophic lateral sclerosis (ALS) [46]. These observations suggest that the CLVS gene family may play an important role in regulating excitatory/inhibitory balance in the brain. Dysregulation of this balance could potentially initiate a cascade of events leading to nervous system diseases [47]. Together, these data support a plausible link between *CLVS2* and AD pathogenesis.

Although we identified *SEPHS2* and *CLVS2* as SCD-related genes, we found limited evidence indicating their association with pathways influencing neurodegenerative diseases. While there is some existing evidence linking *SEPHS2* to neurodegenerative conditions, it is less conclusive than that for *CLVS2*. Despite representing the largest WGS-based rare variant analysis of SCD in the Chinese population to our knowledge, our study marks just the initial step in understanding these two genes. Further investigations to validate the roles of *CLVS2* and *SEPHS2* may provide a more comprehensive exploration of mechanisms related to cognitive decline.

In common variant analysis, we identified genes with suggestive significance. Notably, *SCYL3* has only been previously reported in relation to smoking initiation [48], while *FIRRM* has been linked to aging [49]. Neither gene has been previously associated with cognitive decline. Additionally, we replicated variants associated with cognitive decline, including rs17111203, rs144191744, and rs4488224. These three variants were previously identified in a small-sample-size genetic association study of SCD in a migraine background [9], and were mapped to *TGFBR3*, *PSMA2P1*, and *RNU6-1135P*, respectively. According to the GTEx Portal, transforming growth factor beta receptor 3 (*TGFBR3*) was highly expressed in tissues like the ovary, esophagus, and adipose, with relatively lower expression in the brain. *PSMA2P1* and *RNU6-1135P* exhibited high expression in the artery and adipose tissues; however, their roles in cognitive decline, warrant further investigation.

Although we didn’t detect a significant genetic association between SCD and AD through LDSC, we replicated previously reported AD-related genes *ABCA7* and *CASS4* [19]. *ABCA7* is considered an important genetic determinant for late-onset AD by regulating several molecular processes such as cholesterol metabolism and amyloid processing and clearance [50]. *CASS4* encodes a signaling protein involved in neuritic plaque burden, neurofibrillary tangle formation, and disruption of synaptic connections in AD [51]. Epidemiological and clinical studies have shown that SCD can be an early indicator of AD, but their genetic connections remain underexplored. Our results provide additional genetic insights into the relationship between SCD and AD.

This study utilized two independent cohorts. Although the validation cohort had a smaller sample size (*n* = 1479), which could reduce the power to detect true associations, our findings were largely consistent across both discovery and validation sets for common and rare variant analyses. Future larger, multi-center studies are warranted to reinforce the reliability and robustness of these results.

This study has several strengths. It is the first to apply WGS to SCD in over 10,000 Chinese participants. To our knowledge, it is the largest study on SCD to date in terms of sample size. The scale and methodology facilitate a deeper understanding of the genetic underpinnings of SCD. Additionally, the use of the STAARpipeline enables an exhaustive examination of both rare coding and non-coding variants associated with SCD, leveraging its ability to adjust for covariates and incorporate multiple *in silico* variant functional annotation scores to improve analytical power. Moreover, internal replication and corroborative multi-omics data further strengthen the validity of our findings. Finally, we employed MR analysis to validate *SEPHS2* in individuals of European ancestry, addressing the limitation of lacking external validation to some extent.

We also acknowledge several limitations in our study. First, our discovery cohort and internal validation cohort were processed in two batches. An external validation may further benefit the current study, and we expect future cohort studies to include SCD as one of the phenotypes to facilitate external validation. Second, the study population was from the Chinese-based HOPE Cohort, which limits the generalizability of our findings to other populations. Third, our validation dataset has a smaller sample size, which may reduce its statistical power. Fourth, we used a generalized linear mixed-effects model to analyze bounded count data, the AD8 score, which may not fully account for the upper limit of the response variable.

## Conclusion

In conclusion, we validated cognitive decline-related variants in the HOPE Cohort and identified novel genes associated with SCD, *SEPHS2* and *CLVS2*, supported by multi-omics evidence. This study provides insights into the pathogenic mechanisms and potential therapeutic targets for neurodegenerative diseases.

## Materials and methods

### Study population

This study was conducted based on the ongoing HOPE Cohort in China. The HOPE Cohort is a newly established community-based prospective cohort enrolling participants from both urban and rural areas in Zhejiang Province, China. It has recruited over 100,000 participants from June 2021 to July 2024. Blood and serum specimens from the participants were collected at the enrollment. We included a total of 10,763 participants with WGS data in this study, with 9284 samples assigned to the discovery cohort and 1479 samples to the replication cohort. The samples were processed in different batches, with all discovery samples processed in 2021 and the replication samples processed in 2023 as the cohort progressed. As part of the HOPE Cohort, we included 1007 participants who completed the Montreal Cognitive Assessment (MoCA) in 2023 as a validation set to further confirm our genetic findings.

### Phenotype measurement

In the HOPE Cohort, SCD was measured by the AD8 questionnaire. The AD8 is a brief questionnaire used to screen for cognitive impairment and early mild dementia in the general population [52]. The Chinese version of the AD8 has been validated to efficiently recognize patients with mild cognitive impairment in Taiwan, China [53]. The AD8 questionnaire scores, which varied from 0 to 8 based on the number of positive answers, were subjective measures of participants’ cognitive impairment [52]. In this study, we utilized the continuous AD8 score as the response variable, where a higher score indicates worse cognitive performance.

Additionally, participants who completed the Beijing version of MoCA [54] were selected as an internal validation. MoCA is a widely used tool to screen for MCI. After adjusting for age and years of education, MCI was defined as a MoCA score below 26 [54]. This resulted in 706 MCI cases and 301 controls, all of whom were included in the rare variant analysis for validation as a response variable.

### DNA sample preparation and WGS

Genomic DNA was extracted from peripheral blood samples of the study population using QIAamp 96 DNA QIAcube HT Kit (Catalog No. 51331, QIAGEN, Hilden, Germany). DNA libraries were constructed through MGIEasy FS DNA Library Prep Set (Catalog No. 1000006988, MGI, Shenzhen, China). WGS was performed using the DIPSEQ platform (BGI, Shenzhen, China). To ensure the sequencing quality, samples were excluded based on the following criteria: (1) contamination rate > 5%; (2) duplication rate > 10%; (3) regions with 5× coverage < 80%; or (4) sex check failure.

### Variant calling and QC

We followed the GATK Best Practices pipeline to discover germline short variants including SNPs and indels of 50 bp or less for sequencing alignment [55]. Joint calling was implemented by GATK GenotypeGVCFs. Variants of excess heterozygosity were filtered out. Based on quality scores including QualByDepth (QD), MappingQualityRankSumTest (MQRankSum), ReadPosRankSumTest (ReadPosRankSum), FisherStrand (FS), StrandOddsRatio (SOR), and depth (DP), Variant Quality Score Recalibration (VQSR) was conducted to identify the low-quality variants. To improve the genotyping quality, refinement was performed using Beagle 5.4 [56,57]. Variants that had a Hardy–Weinberg equilibrium (HWE) *P* < 1E−06 were excluded. Potentially monozygotic twins or duplicated samples were also removed from the analysis.

### GWAS

PCA was performed to estimate the genetic background. A sparse genomic relationship matrix (GRM) was estimated using genome-wide complex trait analysis (GCTA) [40]. Biallelic autosome variants were included in the following GWAS analysis. We applied a generalized linear mixed-effects model (fastGWA-GLMM), adjusting for sex, age, and the first 20 principal components [58,59]. To evaluate potential inflation in the association test statistics, we calculated the genomic inflation factor (λ) using the median Chi-squared statistic from the GWAS results. A λ value close to 1 indicates that there is no inflation in the test statistics. Quantile-quantile (QQ) plot and Manhattan plots were constructed in R (v3.1.0; https://www.r-project.org) using the “qqman” package. The genomic inflation factor was calculated in R using the “GenABEL” package. Statistical significance and suggestive significance for the single-variant association analyses were defined as *P* < 5E−08 and *P* < 1E−05, respectively.

### Rare variant association analysis

We followed the variant set test to detect possible rare variant associations using STAARpipeline [60]. Analyses were categorized into gene-centric and non-gene-centric frameworks based on functional annotations. The STAARpipeline utilized nine annotation principal components (aPCs) and three integrative scores including Combined Annotation Dependent Depletion (CADD) [61], Linear Inference of Natural Selection from Interspersed Genomically coHerent elemenTs (LINSIGHT) [62], and Functional Analysis through Hidden Markov Model with eXtended Features (FATHMM-XF) [63] as weights for constructing the variant set association test. The aPCs were derived from the first principal component of individual functional annotation scores, which assess similar biological functions. An omnibus test in the STAARpipeline (STAAR-O) was reported in our main results, which integrates the *P* values of the sequence kernel association test (SKAT), the burden test, and the aggregated Cauchy association test combining variant-level *P* values (ACAT-V). Conditional analysis was conducted with 455 known loci from the GWAS Catalog.

For each protein-coding gene, gene-centric coding analysis was performed for seven categories to aggregate rare coding variants: (1) putative loss-of-function (stop-gain, stop-loss, and splice), (2) missense, (3) disruptive missense, (4) putative loss-of-function plus disruptive missense, and (5) synonymous. We also included PTV and PTV + D masks in the analysis. The putative loss-of-function, missense, and synonymous rare variants were defined by the reference human genome annotation for The ENCODE Project (GENCODE) Variant Effect Predictor (VEP) categories. For missense rare variants, an additional annotation functional category predicting functionally “disruptive” variants was determined by a meta-analytic support vector machine (MetaSVM), which measures the deleteriousness of missense mutations [24].

Similarly, the gene-centric non-coding analysis was performed for eight genetic categories of regulatory regions to aggregate rare non-coding variants: (1) promoter rare variants overlapping cap analysis of gene expression (CAGE) sites, (2) promoter rare variants overlapping DNase I hypersensitive sites (DHSs), (3) enhancer rare variants overlapping CAGE sites, (4) enhancer rare variants overlapping DHSs, (5) UTR rare variants, (6) upstream region rare variants, (7) downstream region rare variants, and (8) non-coding RNA (ncRNA) rare variants. For non-coding genomes, the enhancer rare variants are defined as rare variants in GeneHancer-predicted regions with the overlap of CAGE sites or DHSs. The UTR, upstream, downstream, and ncRNA rare variants are defined by the GENCODE VEP categories. For the UTR mask, rare variants in both 5′ and 3′ UTRs are included. For the ncRNA mask, the exonic and splicing ncRNA rare variants are included. Detailed definition for each category is provided in the STAARpipeline [26].

We employed Bonferroni-corrected significance thresholds of 5E−07 [0.05/(20,000 × 5)] for rare coding variant analysis and 3.57E−07 [0.05/(20,000 × 7)] for rare non-coding gene-centric variant analysis.

### Gene-based and pathway-based analyses of common variants

Gene-based analysis was conducted using Multi-marker Analysis of GenoMic Annotation (MAGMA) implemented in the Functional Mapping and Annotation of Genetic Associations (FUMA) platform. This analysis employs a multiple regression model to accurately consider linkage disequilibrium between markers and identify effects involving multiple markers. Input SNPs were mapped to 18,720 protein-coding genes. Gene-set and tissue-expression analyses were also performed using FUMA-MAGMA. The MAGMA gene-set analysis assessed the overrepresentation of biological functions based on gene annotations using curated gene sets and Gene Ontology (GO) terms obtained from the Molecular Signature Database (MSigDB; v5.2). Genes and gene sets with Bonferroni-corrected *P*_bon_ < 0.05 were considered significantly enriched. The MAGMA tissue-expression analysis was conducted using data from GTEx (v8) to identify the tissue specificity of the phenotype. All MAGMA analyses were run with default settings (number of available genes = 18,052). Bonferroni-corrected significance threshold was set at 2.77E−06 (*P* = 0.05/18,052) for the gene-level association test and 3.13E−06 (*P* = 0.05/15,978) for the GSEA.

### MR analysis

#### Exposures: IVs for discovered genes associated with SCD

In this study, SNPs were selected as IVs to investigate the causal relationship between genes significantly associated with SCD and AD. We obtained a *cis*-expression quantitative trait locus (*cis*-eQTL) dataset from the eQTLGen consortium (https://www.eqtlgen.org/), which assessed the associations between over 11 million variants and the expression levels of 16,989 genes in 31,684 participants across 37 cohorts and 3 gene expression platforms to identify variants related to whole-blood gene expression. Genetic variants significantly associated with the expression of the identified SCD genes were extracted from this dataset. The brain *cis*-eQTL data for the identified genes were obtained from the GTEx Portal, and the reference allele frequencies were imputed using data from the 1000 Genomes Project [64]. Subsequently, the “TwoSampleMR” package was used to analyze the related SNPs and select the non-coding *cis*-acting genetic variants highly correlated with the expression of the discovered genes (*P* < 1E−05). Clumping was conducted with a linkage disequilibrium threshold of *r*^2^ < 0.05 and a clumping distance threshold of 500 kb.

#### Outcome: AD dataset for MR analysis

We retrieved the outcome data of AD, with 954 AD cases and 487,331 controls of European ancestry from the IEU OpenGWAS project (dataset ID: ieu-b-5067; https://gwas.mrcieu.ac.uk/terms).

After determining the IVs, we applied five MR methods: inverse variance weighting, MR-Egger, weighted median, simple mode, and weighted mode. MR-Egger analysis was utilized to assess SNP pleiotropy. Additionally, Cochran’s Q statistic and Rucker’s Q statistic were used to detect heterogeneity. Finally, a leave-one-out analysis was performed to assess the sensitivity of individual SNPs to the overall results.

### LDSC

LDSC was used to assess the genetic correlation between SCD and AD. LDSC is a command-line tool for estimating heritability and genetic correlation from GWAS summary statistics [65,66]. For this analysis, the GWAS summary statistics for SCD were derived from the discovery cohort with a sample size of 9284 individuals. The AD summary statistics were based on a Japanese sample, including 3962 AD cases and 4074 controls [67]. We used East Asian LD scores from the 1000 Genomes Project for this analysis [68].

### Data sources for transcriptomic and phenotypic evidence

The transcriptomics data were sourced from GTEx and The Human Protein Atlas. The association evidence between transcriptomic data and neurodegenerative diseases was obtained from PhenomeXcan, and phenotypic information of related genes was obtained from the AstraZeneca PheWAS Portal.

## Ethical statement

The HOPE Cohort study was approved by the Ethics Committee of the Second Affiliated Hospital of School of Medicine, Zhejiang University, China (Approval Nos. 2020LSYD1013 and 2022LSYD0805). All participants provided informed consent before enrollment.

## Code availability

The code has been submitted to BioCode at the National Genomics Data Center (NGDC), China National Center for Bioinformation (CNCB) (BioCode: BT007601), which is publicly accessible at https://ngdc.cncb.ac.cn/biocode/tools/BT007601.

## Supplementary Material

qzaf063_Supplementary_Data

## Data Availability

The GWAS data supporting this study have been deposited in the Genome Variation Map at the NGDC, CNCB (GVM: GVP000046), which are publicly available at https://ngdc.cncb.ac.cn/gvm/.

## CRediT author statement

**Mengying Wang:** Investigation, Methodology, Data curation, Formal analysis, Software, Writing – original draft, Writing – review & editing. **Liyang Sun:** Investigation, Formal analysis, Project administration, Writing – review & editing. **Xin Xu:** Methodology, Data curation, Supervision. **Ruoqi Dai:** Data curation, Formal analysis, Software. **Qilong Tan:** Methodology, Data curation, Formal analysis, Software. **Yun Zhu:** Data curation, Formal analysis. **Andi Xu:** Investigation, Methodology, Data curation. **Weifang Zheng:** Investigation, Writing – review & editing. **Yuanxing Tu:** Investigation, Writing – review & editing. **Dan Zhou:** Methodology, Data curation, Supervision, Writing – review & editing. **Wenyuan Li:** Methodology, Data curation, Supervision, Writing – review & editing, Validation. **Xifeng Wu:** Conceptualization, Funding acquisition, Investigation, Project administration, Resources, Supervision, Writing – review & editing. All authors have read and approved the final manuscript.
