# Supplementary material for: Whole-genome Sequence Analysis Revealed Novel Subjective Cognitive Decline-associated Genes in 10,763 Chinese
Source: Genomics Proteomics Bioinformatics. 2025 Jul 29;23(5):qzaf063. doi: 10.1093/gpbjnl/qzaf063 (PMC12561000; doi:10.1093/gpbjnl/qzaf063)
Supplement: qzaf063_Supplementary_Data [file qzaf063_supplementary_data.zip › supplementary material captions.docx]

**Supplementary material**

**Figure S1 General description of common variants analysis**

**A.** Manhatton plot from single variant association analysis in discovery dataset; **B.** Manhatton plot from single variant association analysis in the validation dataset; **C.** The QQ plot from single variant association analysis in the discovery dataset; **D.** The QQ plot from single variant association analysis in the validation dataset. QQ, quantile-quantile.

**Figure S2 Manhattan plot of PTV and PTV+D from the discovery dataset**

The red dashed line represents the threshold of *P* = 5E−07. PTV, protein-truncating variants; PTV + D, PTV and disruptive missense variants.

**Figure S3 Expression of gene *SEPHS* and *CLVS* families in bulk tissue**

**A.** Expression of gene *SEPHS2* in bulk tissue; **B.** Expression of gene *SEPHS1* in bulk tissue; **C.** Expression of gene *CLVS2* in bulk tissue; **D**. Expression of gene *CLVS1* in bulk tissue. Expressions were shown in TPM. Data originated from GTEx v8 (dbGaP accession phs000424.v8.p2). TPM, transcripts per million.

**Figure S4 Single-cell type specificity for RNA expression of *SEPHS2* and *CLVS2***

**A.** Single-cell type specificity for RNA expression of gene *SEPHS2*. **B.** Single-cell type specificity for RNA expression of gene *CLVS2*. Data originated from The Human Protein Atlas.

**Table S1 Suggestive significant SNPs in discovery with validation results**

**Table S2 Top 20 genes selected in discovery stage via gene-based analysis with results in validation**

**Table S3 Top 20 gene sets in discovery stage and their performance in validation stage**

**Table S4 455 cognitive decline related loci from the GWAS Catalog**

**Table S5 Candidate genes of AD with results through gene-based analysis**

**Table S6 Characteristics for rare variants accounting for *CLVS2* missense**

**Table S7 Internal validation of rare variant sets in non-coding regions of *SEPHS2* and coding regions of *CLVS2* in MCI cases and controls**

**Table S8 Cognitive decline-related loci after pruning**

**Table S9 Suggestive SCD-related genes from rare variants analysis**

**Table S10 Instrumental variables for Mendelian randomization analysis**

Sheet1: Table S10A, IVs–blood; Sheet2, Table S10A, IVs–brain cerebellum; Sheet3, Table S10A, IVs–brain cerebellar hemisphere.

**Table S11 Proteomic changes for the clavesin and selenophosphate synthetase family in AD brains from a meta-analysis of seven proteomics datasets**

**Table S12 Transcriptomics changes of *CLVS2* and *SEPHS2* in brain tissues of patients with neurodegenerative diseases**

**Table S13 Most significant phenotype (continuous traits) associated with the *CLVS* and *SEPHS* family**
