# Supplementary material for: Whole-genome Sequence Analysis Revealed Novel Subjective Cognitive Decline-associated Genes in 10,763 Chinese
Source: Genomics Proteomics Bioinformatics. 2025 Jul 29;23(5):qzaf063. doi: 10.1093/gpbjnl/qzaf063 (PMC12561000; doi:10.1093/gpbjnl/qzaf063)
Supplement: qzaf063_Supplementary_Data [file qzaf063_supplementary_data.zip › Supplementary table 1.docx]

| **Table S1 Suggestive significant SNPs in discovery with validation results** | | | | | | | | | | | | | | |
| --- | --- | --- | --- | --- | --- | --- | --- | --- | --- | --- | --- | --- | --- | --- |
| **Chr** | **Pos** | **SNP ID** | **Annotation** | **Nearest gene** | **A1** | **A2** | **Discovery** | | | | **Validation** | | | |
|  |  |  |  |  |  |  | **AF** | **Beta** | **SE** | ***P*** | **AF** | **Beta** | **SE** | ***P*** |
| 1 | 1,655,484 | rs144413330 | exonic | *CDK11B* | T | A | 0.017 | 0.504 | 0.106 | 2.08E−06 | 0.014 | 0.202 | 0.284 | 0.477 |
| 1 | 30,451,124 | rs76856684 | intergenic | *MATN1* | G | A | 0.040 | 0.310 | 0.070 | 9.53E−06 | 0.046 | −0.017 | 0.154 | 0.913 |
| 1 | 181,138,484 | rs146543352 | intergenic | *LINC01732* | G | C | 0.066 | 0.250 | 0.056 | 7.99E−06 | 0.064 | −0.239 | 0.138 | 0.082 |
| 4 | 98,546,963 | rs73832344 | intronic | *TSPAN5* | C | T | 0.027 | 0.379 | 0.085 | 8.72E−06 | 0.024 | 0.403 | 0.218 | 0.064 |
| 4 | 129,526,824 | rs56004361 | intergenic | *LINC02466* | A | G | 0.126 | 0.189 | 0.041 | 4.92E−06 | 0.130 | −0.024 | 0.099 | 0.812 |
| 4 | 129,552,538 | rs2138662 | intergenic | *LINC02466* | A | G | 0.135 | 0.179 | 0.040 | 7.89E−06 | 0.140 | −0.015 | 0.097 | 0.879 |
| 4 | 129,553,323 | rs6832617 | intergenic | *LINC02466* | G | A | 0.140 | 0.196 | 0.040 | 7.13E−07 | 0.145 | −0.038 | 0.095 | 0.693 |
| 4 | 129,565,716 | rs62309490 | intergenic | *LINC02466* | A | C | 0.138 | 0.193 | 0.040 | 1.28E−06 | 0.144 | −0.063 | 0.095 | 0.511 |
| 4 | 129,568,985 | rs11935537 | intergenic | *LINC02466* | G | C | 0.139 | 0.193 | 0.040 | 1.12E−06 | 0.144 | −0.076 | 0.095 | 0.424 |
| 4 | 129,589,178 | rs62309518 | intergenic | *LINC02466* | C | T | 0.146 | 0.183 | 0.039 | 2.58E−06 | 0.151 | −0.076 | 0.094 | 0.421 |
| 4 | 129,598,767 | rs12648214 | intergenic | *LINC02466* | C | T | 0.139 | 0.195 | 0.040 | 8.84E−07 | 0.144 | −0.063 | 0.095 | 0.511 |
| 4 | 129,608,822 | rs62309528 | intergenic | *LINC02466* | T | A | 0.139 | 0.194 | 0.040 | 1.03E−06 | 0.144 | −0.036 | 0.095 | 0.703 |
| 4 | 129,609,478 | rs12649182 | intergenic | *LINC02466* | T | C | 0.139 | 0.193 | 0.040 | 1.23E−06 | 0.144 | −0.036 | 0.095 | 0.703 |
| 4 | 129,622,555 | rs16998016 | intergenic | *LINC02466* | T | C | 0.139 | 0.186 | 0.040 | 2.72E−06 | 0.144 | −0.036 | 0.095 | 0.703 |
| 4 | 129,634,333 | rs17050575 | intergenic | *LINC02466* | G | A | 0.137 | 0.194 | 0.040 | 1.26E−06 | 0.143 | −0.047 | 0.096 | 0.625 |
| 5 | 2,630,537 | rs74610115 | intergenic | *LSINCT5* | G | A | 0.074 | −0.241 | 0.052 | 3.94E−06 | 0.063 | 0.175 | 0.135 | 0.194 |
| 5 | 2,632,196 | rs75279790 | intergenic | *LSINCT5* | C | A | 0.074 | −0.239 | 0.052 | 4.58E−06 | 0.063 | 0.185 | 0.135 | 0.170 |
| 5 | 2,632,575 | rs77182791 | intergenic | *LSINCT5* | C | G | 0.074 | −0.249 | 0.052 | 1.93E−06 | 0.063 | 0.185 | 0.135 | 0.170 |
| 5 | **135,109,292** | **rs254559** | **ncRNA_intronic** | ***C5orf66*** | **A** | **C** | **0.198** | **0.152** | **0.034** | **8.96E−06** | **0.219** | **−0.167** | **0.081** | **0.040** |
| 6 | 147,931,423 | rs662087 | intergenic | *SASH1* | C | T | 0.487 | −0.122 | 0.027 | 8.33E−06 | 0.489 | 0.023 | 0.066 | 0.722 |
| 6 | 147,945,617 | rs6919109 | intergenic | *SASH1* | T | C | 0.514 | 0.122 | 0.027 | 7.80E−06 | 0.513 | −0.014 | 0.065 | 0.831 |
| 7 | 72,615,058 | rs149352734 | intronic | *TYW1B* | G | A | 0.020 | −0.439 | 0.098 | 7.43E−06 | 0.019 | 0.207 | 0.242 | 0.392 |
| 8 | 132,629,335 | rs117761088 | intronic | *LRRC6* | T | C | 0.024 | 0.407 | 0.091 | 7.12E−06 | 0.020 | 0.332 | 0.238 | 0.163 |
| 13 | 94,756,163 | rs139291680 | intergenic | *LOC101927284* | T | C | 0.016 | −0.504 | 0.111 | 5.97E−06 | 0.016 | −0.252 | 0.263 | 0.336 |
| 15 | 99,954,048 | rs79270377 | intergenic | *ADAMTS17* | G | C | 0.107 | 0.212 | 0.045 | 2.19E−06 | 0.099 | −0.057 | 0.111 | 0.605 |
| 17 | 65,184,197 | rs139147156 | intronic | *RGS9* | C | T | 0.013 | 0.555 | 0.121 | 4.73E−06 | 0.011 | −0.163 | 0.316 | 0.605 |
| 20 | 46,530,963 | rs6512382 | intergenic | *OCSTAMP* | C | T | 0.271 | −0.138 | 0.031 | 9.21E−06 | 0.273 | −0.010 | 0.075 | 0.894 |
| 22 | 47,900,193 | rs76355438 | intergenic | *LOC284930* | T | C | 0.073 | 0.252 | 0.053 | 1.83E−06 | 0.072 | −0.131 | 0.127 | 0.303 |
| 22 | 47,901,215 | rs75133479 | intergenic | *LOC284930* | T | A | 0.072 | 0.239 | 0.053 | 6.20E−06 | 0.071 | −0.118 | 0.128 | 0.357 |
| 22 | 47,904,412 | rs76524991 | intergenic | *LOC284930* | G | A | 0.073 | 0.256 | 0.052 | 1.11E−06 | 0.073 | −0.100 | 0.127 | 0.429 |
| 22 | 47,911,020 | rs74557385 | intergenic | *LOC284930* | G | A | 0.073 | 0.250 | 0.053 | 2.17E−06 | 0.072 | −0.136 | 0.127 | 0.285 |
| 22 | 47,918,782 | rs5766940 | intergenic | *LOC284930* | C | G | 0.051 | 0.284 | 0.062 | 5.07E−06 | 0.048 | −0.018 | 0.155 | 0.908 |
| 22 | 47,920,388 | rs5768159 | intergenic | *LOC284930* | A | G | 0.061 | 0.255 | 0.057 | 8.52E−06 | 0.057 | −0.147 | 0.145 | 0.311 |
| 22 | 47,920,933 | rs5768161 | intergenic | *LOC284930* | C | G | 0.060 | 0.261 | 0.058 | 5.82E−06 | 0.057 | −0.119 | 0.144 | 0.410 |
| 22 | 47,921,855 | rs12628536 | intergenic | *LOC284930* | A | G | 0.060 | 0.259 | 0.057 | 6.64E−06 | 0.057 | −0.119 | 0.144 | 0.410 |
| 22 | 47,921,861 | rs6008455 | intergenic | *LOC284930* | A | G | 0.060 | 0.261 | 0.057 | 5.48E−06 | 0.057 | −0.119 | 0.144 | 0.410 |
| 22 | 47,921,865 | rs6008456 | intergenic | *LOC284930* | C | T | 0.060 | 0.260 | 0.058 | 6.09E−06 | 0.057 | −0.119 | 0.144 | 0.410 |
| 22 | 47,921,925 | rs6008458 | intergenic | *LOC284930* | G | T | 0.060 | 0.261 | 0.058 | 5.66E−06 | 0.057 | −0.119 | 0.144 | 0.410 |
| 22 | 47,921,946 | rs35520788 | intergenic | *LOC284930* | G | C | 0.060 | 0.261 | 0.058 | 5.63E−06 | 0.057 | −0.119 | 0.144 | 0.410 |
| 22 | 47,922,449 | rs5768165 | intergenic | *LOC284930* | T | G | 0.060 | 0.267 | 0.058 | 3.69E−06 | 0.057 | −0.119 | 0.144 | 0.410 |
| 22 | 47,923,096 | rs7292145 | intergenic | *LOC284930* | A | G | 0.060 | 0.260 | 0.058 | 6.53E−06 | 0.057 | −0.112 | 0.145 | 0.438 |
| 22 | 47,923,286 | rs5768167 | intergenic | *LOC284930* | T | C | 0.060 | 0.261 | 0.058 | 6.05E−06 | 0.057 | −0.093 | 0.145 | 0.519 |
| 22 | 47,923,586 | rs5768168 | intergenic | *LOC284930* | C | T | 0.060 | 0.260 | 0.058 | 6.71E−06 | 0.056 | −0.104 | 0.145 | 0.474 |
| 22 | 47,923,715 | rs5768169 | intergenic | *LOC284930* | C | G | 0.060 | 0.261 | 0.058 | 6.31E−06 | 0.056 | −0.104 | 0.145 | 0.474 |
| 22 | 47,924,299 | rs78765720 | intergenic | *LOC284930* | G | A | 0.060 | 0.260 | 0.058 | 6.71E−06 | 0.056 | −0.104 | 0.145 | 0.474 |
| 22 | 47,924,358 | rs112124262 | intergenic | *LOC284930* | C | A | 0.060 | 0.260 | 0.058 | 6.71E−06 | 0.056 | −0.104 | 0.145 | 0.474 |
| 22 | 47,925,727 | rs5768173 | intergenic | *LOC284930* | T | A | 0.060 | 0.260 | 0.058 | 6.65E−06 | 0.056 | −0.104 | 0.145 | 0.474 |

*Note*: SNPs with MAF >= 0.01 in both discovery and validation dataset were selected and ranked by *P* value in discovery dataset. SNPs with *P* value < 1E−05 in discovery datasets were listed. MAF, minor allele frequency.
