# Supplementary material for: Whole-genome Sequence Analysis Revealed Novel Subjective Cognitive Decline-associated Genes in 10,763 Chinese
Source: Genomics Proteomics Bioinformatics. 2025 Jul 29;23(5):qzaf063. doi: 10.1093/gpbjnl/qzaf063 (PMC12561000; doi:10.1093/gpbjnl/qzaf063)
Supplement: qzaf063_Supplementary_Data [file qzaf063_supplementary_data.zip › Supplementary table 11.docx]

| **Table** **S11 Proteomic changes for the clavesin and selenophosphate synthetase family in AD brains from a meta-analysis of seven proteomics datasets** | | | | | | | | | | |
| --- | --- | --- | --- | --- | --- | --- | --- | --- | --- | --- |
| **Gene name** | **Protein accession** | **Annotation** | **Direction in one-tailed *t*-tests** | **Fisher's combined *P*** | **Median of log_2_(AD/control)** | **Cell type expression by RNA-seq** | | | | |
|  |  |  |  |  |  | **Astrocytes** | **Neurons** | **Oligodendrocytes** | **Microglia** | **Endothelia** |
|  |  |  |  |  |  |  |  |  |  |  |
| *SEPHS2* | sp\|Q99611\|SPS2_HUMAN | Selenide, water dikinase 2 | Down in AD | 6.92E−02 | −0.01 | 3.74 | 4.49 | 2.68 | 3.19 | 1.70 |
| *SEPHS1* | sp\|P49903\|SPS1_HUMAN | Selenide, water dikinase 1 | Up in AD | 9.56E−02 | 0.04 | 3.35 | 4.06 | 2.58 | 2.76 | 1.72 |
| *CLVS2* | sp\|Q5SYC1\|CLVS2_HUMAN | Clavesin-2 | Down in AD | 8.75E−03 | −0.02 | 2.15 | 5.07 | 0.26 | 0.08 | 0.15 |
| *CLVS1* | sp\|Q8IUQ0\|CLVS1_HUMAN | Clavesin-1 | Down in AD | 8.64E−03 | −0.07 | 0.93 | 5.30 | 2.09 | 0.37 | 1.11 |

*Note*: Meta-analysis of proteomic summary data was integrated by Bai et al, deriving from seven independent proteomic datasets. The ratio numbers of AD case and control were shown in the logarithm. *P* values were calculated using the Fisher’s method. RNA-seq expression in a total of 5 cell types was quantified by the log_2_(TPM + 1) values. For more details, see Bai et al. RNA-seq, RNA sequencing; TPM, transcripts per million.
