# Supplementary material for: Whole-genome Sequence Analysis Revealed Novel Subjective Cognitive Decline-associated Genes in 10,763 Chinese
Source: Genomics Proteomics Bioinformatics. 2025 Jul 29;23(5):qzaf063. doi: 10.1093/gpbjnl/qzaf063 (PMC12561000; doi:10.1093/gpbjnl/qzaf063)
Supplement: qzaf063_Supplementary_Data [file qzaf063_supplementary_data.zip › Supplementary table 12.docx]

| **Table S12 Transcriptomics changes of *CLVS2* and *SEPHS2* in brain tissues of patients with neurodegenerative diseases** | | | | | |
| --- | --- | --- | --- | --- | --- |
| **Gene name** | **Phenotype** | **Tissue** | **Effect size** | **Z-score** | ***P*** |
|  |  |  |  |  |  |
| *CLVS2* | Alzheimer’s disease | Brain putamen basal ganglia | −2.65E−03 | −2.56 | 0.011 |
|  |  | Brain cerebellum | −1.29E−03 | −2.34 | 0.019 |
| *SEPHS2* | Parkinson’s disease | Brain cortex | 0.03 | 2.79 | 0.005 |
|  |  | Brain putamen basal ganglia | 0.02 | 2.79 | 0.005 |
|  |  | Brain frontal cortex BA9 | 0.03 | 2.79 | 0.005 |

*Note*: Transcriptomic data was collected in PhenomeXcan. A total of 3 keywords of neurodegenerative diseases (Alzheimer’s disease, Parkinson’s disease, and dementia) were searched and only tissues related to the brain were selected.
