# Supplementary material for: Whole-genome Sequence Analysis Revealed Novel Subjective Cognitive Decline-associated Genes in 10,763 Chinese
Source: Genomics Proteomics Bioinformatics. 2025 Jul 29;23(5):qzaf063. doi: 10.1093/gpbjnl/qzaf063 (PMC12561000; doi:10.1093/gpbjnl/qzaf063)
Supplement: qzaf063_Supplementary_Data [file qzaf063_supplementary_data.zip › Supplementary table 13.docx]

| **Gene** | **Phenotype (Continuous traits)** | ***P*** | **Effect size** | **CI** |
| --- | --- | --- | --- | --- |
| *SEPHS2* | High light scatter reticulocyte count | 2.30E−05 | 0.18 | 0.09–0.26 |
| *SEPHS1* | Weighted-mean FA in tract acoustic radiation (right) | 1.19E−04 | −1.01 | −1.52–−0.49 |
| *CLVS2* | Volume of grey matter in X cerebellum (vermis) | 2.38E−05 | −0.84 | −1.22–−0.45 |
| *CLVS1* | Volume of grey matter in middle temporal gyrus\| temporooccipital part (right) | 4.39E−06 | −1.02 | −1.45–−0.58 |

**Table 13** **Most significant phenotype (continuous traits) associated with the *CLVS* and *SEPHS* family**

*Note*: Data originated from website AstraZeneca PheWAS Portal (UK Biobank 450k v4 Public). Only results of the most significant continuous traits for each gene were extracted. CI, confidence interval; FA, fractional anisotropy.
