# Supplementary material for: Whole-genome Sequence Analysis Revealed Novel Subjective Cognitive Decline-associated Genes in 10,763 Chinese
Source: Genomics Proteomics Bioinformatics. 2025 Jul 29;23(5):qzaf063. doi: 10.1093/gpbjnl/qzaf063 (PMC12561000; doi:10.1093/gpbjnl/qzaf063)
Supplement: qzaf063_Supplementary_Data [file qzaf063_supplementary_data.zip › Supplementary table 2.docx]

| **Table S2 Top 20 genes selected in discovery stage via gene-based analysis with results in validation** | | | | | | | | | |
| --- | --- | --- | --- | --- | --- | --- | --- | --- | --- |
| **Gene name** | **Chr** | **Start** | **Stop** | **Discovery** | | | **Validation** | | |
|  |  |  |  | **nSNP** | **ZSTAT** | ***P*** | **nSNP** | **ZSTAT** | ***P*** |
| *SCYL3* | 1 | 169,853,074 | 169,894,267 | 120 | 3.95 | 3.91E−05 | 106 | 1.41 | 0.08 |
| *FIRRM* | 1 | 169,795,005 | 169,853,088 | 164 | 3.84 | 6.12E−05 | 127 | 1.63 | 0.052 |
| *PECAM1* | 17 | 64,319,415 | 64,413,801 | 26 | 3.55 | 1.95E−04 | 20 | 0.51 | 0.304 |
| *OR14A16* | 1 | 247,814,800 | 247,834,858 | 2 | 3.5 | 2.29E−04 | 2 | −0.03 | 0.513 |
| *METTL18* | 1 | 169,792,529 | 169,794,959 | 2 | 3.44 | 2.87E−04 | 1 | −0.07 | 0.527 |
| *LRRC63* | 13 | 46,211,911 | 46,276,818 | 132 | 3.37 | 3.82E−04 | 90 | 0.82 | 0.205 |
| *PLAAT2* | 11 | 63,552,759 | 63,565,089 | 12 | 3.3 | 4.90E−04 | 8 | −1.56 | 0.941 |
| *XKR3* | 22 | 16,783,412 | 16,821,694 | 81 | 3.28 | 5.21E−04 | 49 | 0.28 | 0.389 |
| *TMEM273* | 10 | 49,154,724 | 49,188,585 | 50 | 3.22 | 6.30E−04 | 44 | 0.15 | 0.441 |
| *PPP3R2* | 9 | 101,591,615 | 101,595,001 | 4 | 3.22 | 6.50E−04 | 4 | 0.66 | 0.254 |
| *STAG3* | 7 | 100,177,563 | 100,214,387 | 42 | 3.21 | 6.69E−04 | 28 | −1.05 | 0.852 |
| *FSHR* | 2 | 48,866,435 | 49,154,527 | 652 | 3.16 | 7.95E−04 | 528 | 1.18 | 0.118 |
| *SLC14A2* | 18 | 45,212,957 | 45,683,107 | 1001 | 3.12 | 9.10E−04 | 891 | 0.1 | 0.46 |
| *KRCC1* | 2 | 88,027,203 | 88,055,801 | 37 | 3.1 | 9.55E−04 | 29 | −2.6 | 0.995 |
| *STAR* | 8 | 38,142,700 | 38,151,265 | 6 | 3.09 | 9.87E−04 | 5 | 0.09 | 0.465 |
| *RAB11FIP2* | 10 | 118,004,916 | 118,046,877 | 46 | 3.09 | 1.00E−03 | 34 | −0.64 | 0.74 |
| *ZNF234* | 19 | 44,141,530 | 44,160,309 | 22 | 3.05 | 1.13E−03 | 15 | 0.27 | 0.395 |
| *EEFSEC* | 3 | 128,153,457 | 128,426,193 | 220 | 3.05 | 1.16E−03 | 191 | −0.61 | 0.73 |
| *LOC105375433* | 7 | 102,407,507 | 102,421,280 | 10 | 3.05 | 1.16E−03 | 8 | 1.39 | 0.083 |
| *SLC15A4* | 12 | 128,793,194 | 128,823,996 | 88 | 2 | 1.37E−03 | 79 | −0.09 | 0.534 |
