# Supplementary material for: Whole-genome Sequence Analysis Revealed Novel Subjective Cognitive Decline-associated Genes in 10,763 Chinese
Source: Genomics Proteomics Bioinformatics. 2025 Jul 29;23(5):qzaf063. doi: 10.1093/gpbjnl/qzaf063 (PMC12561000; doi:10.1093/gpbjnl/qzaf063)
Supplement: qzaf063_Supplementary_Data [file qzaf063_supplementary_data.zip › Supplementary table 3.docx]

**Table S3 Top 20 gene sets in discovery stage and their performance in validation stage**

| **Gene-set name** | **Discovery** | | | | **Validation** | | | |
| --- | --- | --- | --- | --- | --- | --- | --- | --- |
|  | **nGENES** | **Beta** | **SE** | ***P*** | **nGENES** | **Beta** | **SE** | ***P*** |
| GOBP_IMMUNE_RESPONSE_INHIBITING_SIGNAL_TRANSDUCTION | 10 | 1.073 | 0.267 | 2.89E–05 | 10 | 0.105 | 0.277 | 0.352 |
| REACTOME_DAG_AND_IP3_SIGNALING | 40 | 0.514 | 0.129 | 3.58E–05 | 39 | 0.052 | 0.143 | 0.358 |
| GOBP_CENTRAL_NERVOUS_SYSTEM_MYELIN_MAINTENANCE | 4 | 1.550 | 0.406 | 6.72E–05 | 4 | 0.369 | 0.428 | 0.194 |
| LEIN_PONS_MARKERS | 83 | 0.326 | 0.086 | 8.11E–05 | 81 | –0.036 | 0.093 | 0.652 |
| REACTOME_CAMK_IV_MEDIATED_PHOSPHORYLATION_OF_CREB | 10 | 0.990 | 0.263 | 8.59E–05 | 10 | –0.353 | 0.264 | 0.909 |
| REACTOME_CREB1_PHOSPHORYLATION_THROUGH_THE_  ACTIVATION_OF_CAMKII_CAMKK_CAMKIV_CASCASDE | 8 | 1.067 | 0.284 | 8.60E–05 | 8 | –0.256 | 0.285 | 0.816 |
| MIR3175 | 166 | 0.232 | 0.062 | 1.00E–04 | 165 | –0.058 | 0.060 | 0.834 |
| GSE9960_GRAM_NEG_VS_GRAM_POS_SEPSIS_PBMC_UP | 179 | 0.218 | 0.059 | 1.16E–04 | 177 | –0.032 | 0.060 | 0.703 |
| GOBP_REGULATION_OF_EXOCYTOSIS | 181 | 0.225 | 0.062 | 1.26E–04 | 178 | –0.021 | 0.063 | 0.633 |
| HP_SPINAL_CORD_COMPRESSION | 22 | 0.596 | 0.164 | 1.40E–04 | 21 | 0.139 | 0.179 | 0.220 |
| REACTOME_G2_M_DNA_DAMAGE_CHECKPOINT | 72 | 0.332 | 0.092 | 1.58E–04 | 70 | 0.119 | 0.094 | 0.103 |
| GOMF_MHC_CLASS_I_PROTEIN_COM... | 5 | 1.168 | 0.329 | 1.97E–04 | 5 | –0.397 | 0.357 | 0.867 |
| GOBP_REGULATION_OF_SYNAPTIC_...1 | 166 | 0.218 | 0.062 | 2.04E–04 | 164 | 0.031 | 0.063 | 0.314 |
| REACTOME_G_PROTEIN_MEDIATED_... | 53 | 0.387 | 0.112 | 2.69E–04 | 52 | –0.022 | 0.121 | 0.571 |
| MIR6769A_5P | 44 | 0.423 | 0.122 | 2.72E–04 | 44 | –0.257 | 0.129 | 0.977 |
| BENPORATH_ES_CORE_NINE | 8 | 0.886 | 0.258 | 3.00E–04 | 8 | 0.010 | 0.297 | 0.487 |
| GGGNNTTTCC_NFKB_Q6_01 | 127 | 0.247 | 0.073 | 3.40E–04 | 123 | –0.114 | 0.077 | 0.930 |
| WP_SPHINGOLIPID_METABOLISM_G... | 23 | 0.647 | 0.192 | 3.64E–04 | 22 | 0.058 | 0.185 | 0.377 |
| GSE22886_UNSTIM_VS_STIM_MEMO...2 | 182 | 0.209 | 0.062 | 3.90E–04 | 181 | –0.099 | 0.063 | 0.941 |
| GOMF_SELENOCYSTEINE_INSERTIO... | 5 | 1.336 | 0.399 | 4.04E–04 | 5 | 0.081 | 0.400 | 0.420 |

*Note*: Gene sets were derived from MSigDB (GSEA) while analysis was performed by MAGMA using LD reference panel of East Asian. Top 20 gene sets in discovery stage were selected and their validation results were also shown. nGENE, number of genes; MSigDB, Molecular Signatures Database; GSEA, gene set enrichment analysis; MAGMA, Multi-marker Analysis of GenoMic Annotation; LD, linkage disequilibrium.
