# Supplementary material for: Whole-genome Sequence Analysis Revealed Novel Subjective Cognitive Decline-associated Genes in 10,763 Chinese
Source: Genomics Proteomics Bioinformatics. 2025 Jul 29;23(5):qzaf063. doi: 10.1093/gpbjnl/qzaf063 (PMC12561000; doi:10.1093/gpbjnl/qzaf063)
Supplement: qzaf063_Supplementary_Data [file qzaf063_supplementary_data.zip › Supplementary table 5.docx]

| **Table S5 Candidate genes of AD with results through gene-based analysis** | | | | | | | | | | | | | |
| --- | --- | --- | --- | --- | --- | --- | --- | --- | --- | --- | --- | --- | --- |
| **Gene name** | **Chr** | **Start** | **Stop** | **Discovery** | | | | | **Validation** | | | | |
|  |  |  |  | **nSNP** | **nPARAM** | **N** | **ZSTAT** | ***P*** | **nSNP** | **nPARAM** | **N** | **ZSTAT** | ***P*** |
| *ABCA7* | 19 | 1,040,103 | 1,065,572 | 34 | 8 | 9284 | 2.100 | 0.018 | 20 | 5 | 1479 | 0.089 | 0.464 |
| *CASS4* | 20 | 56,411,548 | 56,459,340 | 59 | 12 | 9284 | 1.653 | 0.049 | 47 | 11 | 1479 | 0.474 | 0.318 |
| *CD33* | 19 | 51,215,537 | 51,240,019 | 27 | 8 | 9284 | 1.567 | 0.059 | 22 | 8 | 1479 | −0.437 | 0.669 |
| *APOE* | 19 | 44,905,749 | 44,909,395 | 1 | 1 | 9284 | 1.223 | 0.111 | NA | NA | NA | NA | NA |
| *CLU* | 8 | 27,596,917 | 27,615,031 | 23 | 6 | 9284 | 1.112 | 0.133 | 18 | 4 | 1479 | −0.174 | 0.569 |
| *SORL1* | 11 | 121,452,203 | 121,633,762 | 228 | 33 | 9284 | 0.855 | 0.196 | 181 | 30 | 1479 | −1.081 | 0.860 |
| *HLA-DRA* | 6 | 32,439,842 | 32,445,046 | 43 | 10 | 9284 | 0.550 | 0.291 | 37 | 10 | 1479 | −2.604 | 0.995 |
| *BIN1* | 2 | 127,048,023 | 127,107,400 | 147 | 25 | 9284 | 0.370 | 0.356 | 111 | 21 | 1479 | 0.164 | 0.435 |
| *MS4A6A* | 11 | 60,171,607 | 60,184,666 | 19 | 4 | 9284 | 0.269 | 0.394 | 18 | 3 | 1479 | −0.337 | 0.632 |
| *ADAM10* | 15 | 58,595,204 | 58,749,978 | 184 | 17 | 9284 | 0.132 | 0.447 | 120 | 14 | 1479 | −1.196 | 0.884 |
| *EPHA1* | 7 | 143,390,782 | 143,408,892 | 28 | 9 | 9284 | 0.048 | 0.481 | 25 | 9 | 1479 | 1.340 | 0.090 |
| *PILRA* | 7 | 100,373,445 | 100,400,099 | 16 | 4 | 9284 | 0.048 | 0.481 | 13 | 3 | 1479 | 0.507 | 0.306 |
| *SCIMP* | 17 | 5,208,920 | 5,234,860 | 57 | 11 | 9284 | −0.519 | 0.698 | 48 | 10 | 1479 | 0.617 | 0.269 |
| *CD2AP* | 6 | 47,477,746 | 47,627,263 | 197 | 12 | 9284 | −0.718 | 0.764 | 125 | 10 | 1479 | 0.535 | 0.296 |
| *PTK2B* | 8 | 27,311,482 | 27,459,391 | 296 | 22 | 9284 | −1.052 | 0.854 | 240 | 25 | 1479 | −1.260 | 0.896 |
| *FERMT2* | 14 | 52,857,271 | 52,951,097 | 123 | 14 | 9284 | −1.220 | 0.889 | 86 | 12 | 1479 | 0.149 | 0.441 |
| *PICALM* | 11 | 85,957,171 | 86,069,881 | 152 | 14 | 9284 | −1.749 | 0.960 | 104 | 13 | 1479 | −0.017 | 0.507 |

*Note*: Candidate genes were obtained from gene-based results in Jansen et al and known AD loci/genes. We then performed gene-based analysis for these genes in our discovery and validation dataset through MAGMA. nPARAM, number of parameters; MAGMA, multi-marker analysis of genomic annotation.
