# Supplementary material for: Whole-genome Sequence Analysis Revealed Novel Subjective Cognitive Decline-associated Genes in 10,763 Chinese
Source: Genomics Proteomics Bioinformatics. 2025 Jul 29;23(5):qzaf063. doi: 10.1093/gpbjnl/qzaf063 (PMC12561000; doi:10.1093/gpbjnl/qzaf063)
Supplement: qzaf063_Supplementary_Data [file qzaf063_supplementary_data.zip › Supplementary table 6.docx]

| **Table S6**  **Characteristics for rare variants accounting for *CLVS2* missense** | | | | | | | | | | | | | |
| --- | --- | --- | --- | --- | --- | --- | --- | --- | --- | --- | --- | --- | --- |
| **CHR** | **Start** | **Stop** | **Ref** | **Alt** | **Exonic function reference gene** | **AA change reference gene** | **Cyto**  **band** | **SIFT_score** | **SIFT_pred** | **PolyPhen2_HDIV_score** | **PolyPhen2_HDIV_pred** | **PolyPhen2_HVAR_score** | **PolyPhen2_HVAR_pred** |
| 6 | 122,997,892 | 122,997,892 | A | G | nonsynonymous SNV | CLVS2:NM_001010852:exon2:c.A115G:p.I39V | 6q22.31 | 0.328 | T | 0.004 | B | 0.022 | B |
| 6 | 122,997,920 | 122,997,920 | G | T | nonsynonymous SNV | CLVS2:NM_001010852:exon2:c.G143T:p.R48L | 6q22.31 | 0.01 | D | 0.988 | D | 0.927 | D |
| 6 | 123,011,013 | 123,011,013 | A | G | nonsynonymous SNV | CLVS2:NM_001010852:exon3:c.A418G:p.I140V | 6q22.31 | 0.138 | T | 0.197 | B | 0.101 | B |
| 6 | 123,011,031 | 123,011,031 | G | A | nonsynonymous SNV | CLVS2:NM_001010852:exon3:c.G436A:p.A146T | 6q22.31 | 0.406 | T | 0.019 | B | 0.021 | B |
| 6 | 123,011,110 | 123,011,110 | C | T | nonsynonymous SNV | CLVS2:NM_001010852:exon3:c.C515T:p.A172V | 6q22.31 | 0.109 | T | 0.994 | D | 0.953 | D |
| 6 | 123,063,709 | 123,063,709 | G | A | nonsynonymous SNV | CLVS2:NM_001010852:exon6:c.G932A:p.R311H | 6q22.31 | 0.891 | T | 0.001 | B | 0 | B |
| *Note*: Exonic variant function annotation results were generated by ANNOVAR in hg38. Exonic function, AA change and cytoband of variants were showed. SIFT and PolyPhen-2 scores were calculated. SIFT_pred: T = tolerated, D = damaging; PolyPhen2_HVAR_pred or PolyPhen2_HDIV_pred: B = benign, D = damaging. AA, amino acid; SIFT, Sorting Intolerant From Tolerant; PolyPhen-2, Polymorphism Phenotyping v2. | | | | | | | | | | | | | |
