# Supplementary material for: Whole-genome Sequence Analysis Revealed Novel Subjective Cognitive Decline-associated Genes in 10,763 Chinese
Source: Genomics Proteomics Bioinformatics. 2025 Jul 29;23(5):qzaf063. doi: 10.1093/gpbjnl/qzaf063 (PMC12561000; doi:10.1093/gpbjnl/qzaf063)
Supplement: qzaf063_Supplementary_Data [file qzaf063_supplementary_data.zip › Supplementary table 7.docx]

**Table S7 Internal validation of rare variant sets in non-coding regions of *SEPHS2* and coding regions of *CLVS2* in MCI cases and controls**

| **Gene name** | **Category** | **Validation (MCI)** | | | | |
| --- | --- | --- | --- | --- | --- | --- |
|  |  | **nSNV** | **SKAT** | **Burden** | **ACAT-V** | **STAAR-O** |
| *SEPHS2* | Upstream | 6 | 0.599 | 0.722 | 0.73 | **0.847** |
|  | Downstream | 4 | 0.339 | 0.871 | 0.871 | **0.839** |
|  | Enhancer_CAGE | 5 | 0.013 | 0.009 | 0.01 | **0.012** |
|  | Enhancer_DHS | 13 | 0.024 | 0.039 | 0.043 | **0.029** |
|  | Promoter_CAGE | 5 | 0.012 | 0.009 | 0.01 | **0.012** |
|  | Promoter_DHS | 17 | 0.039 | 0.14 | 0.15 | **0.062** |
|  | UTR | 6 | 0.531 | 0.395 | 0.397 | **0.43** |
| *CLVS2* | Missense | / | / | / | / | / |
|  | Synonymous | / | / | / | / | / |

*Note*: Results of rare variant sets were performed applying STAAR pipeline, and *P* values in STAAR-O were finally used in identifying associations. MCI, mild cognitive decline.
