# Supplementary figures and images for: Whole-genome Sequence Analysis Revealed Novel Subjective Cognitive Decline-associated Genes in 10,763 Chinese
Source: Genomics Proteomics Bioinformatics. 2025 Jul 29;23(5):qzaf063. doi: 10.1093/gpbjnl/qzaf063 (PMC12561000; doi:10.1093/gpbjnl/qzaf063)

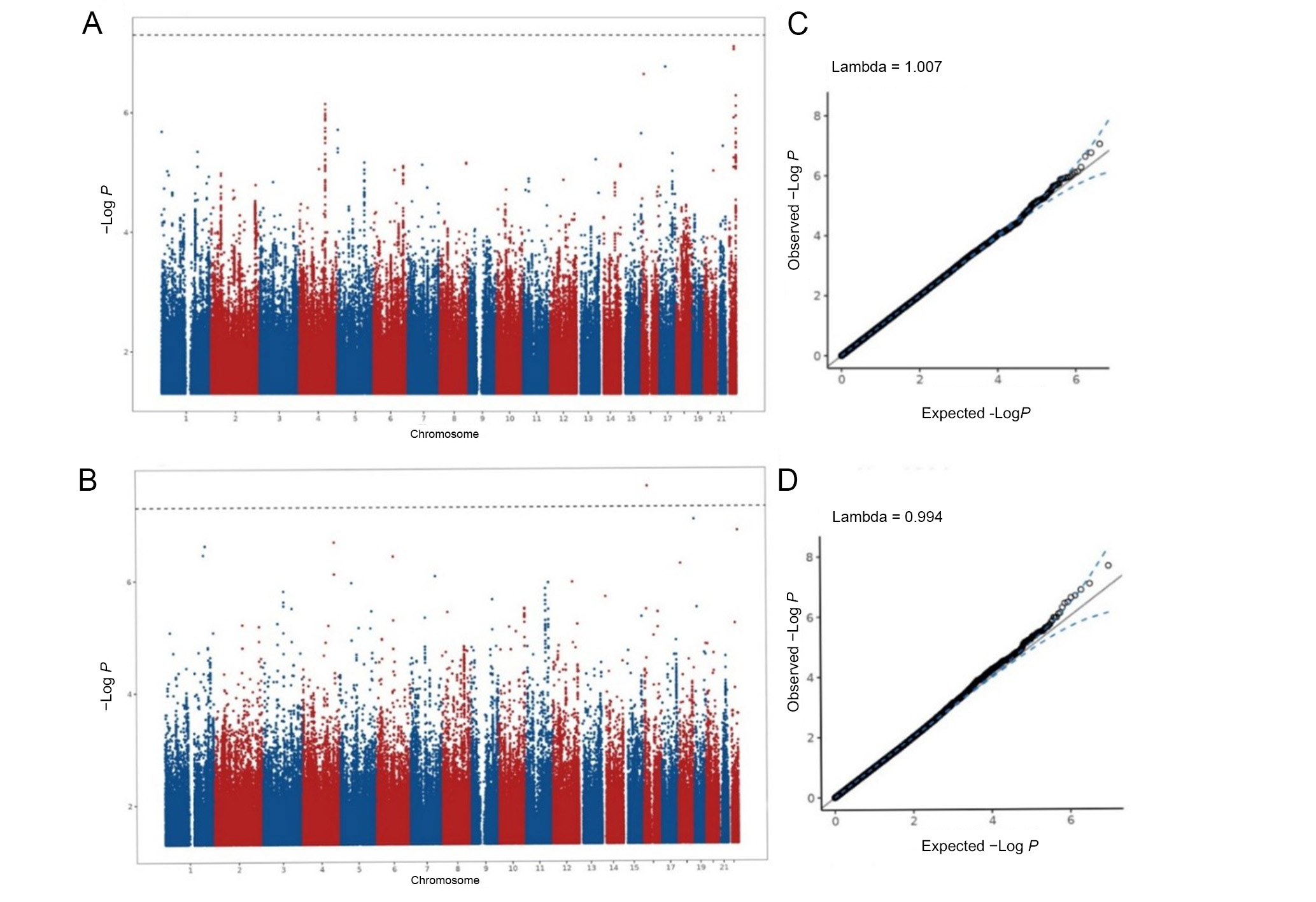

Supplement: qzaf063_Supplementary_Data [file qzaf063_supplementary_data.zip › Supplementary figure 1.jpg]

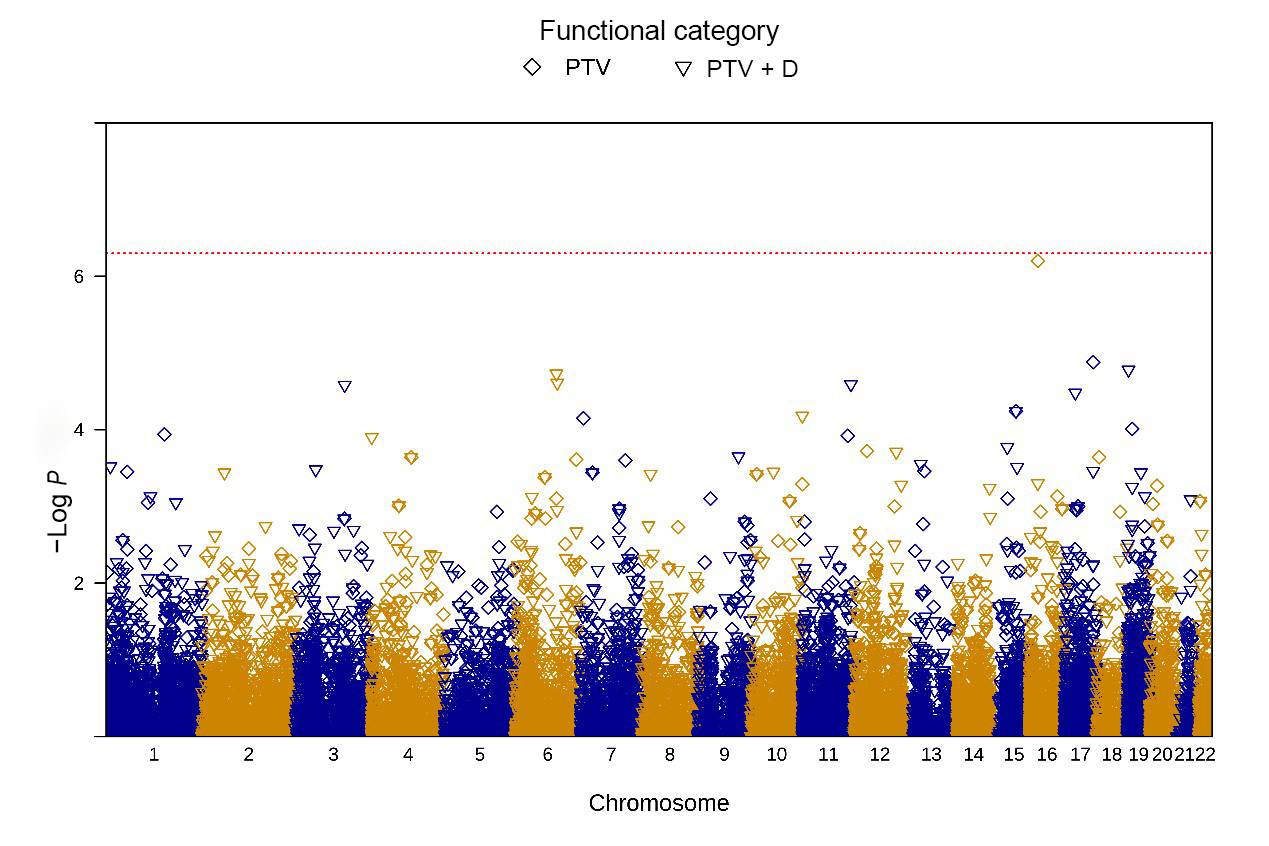

Supplement: qzaf063_Supplementary_Data [file qzaf063_supplementary_data.zip › Supplementary figure 2.jpg]

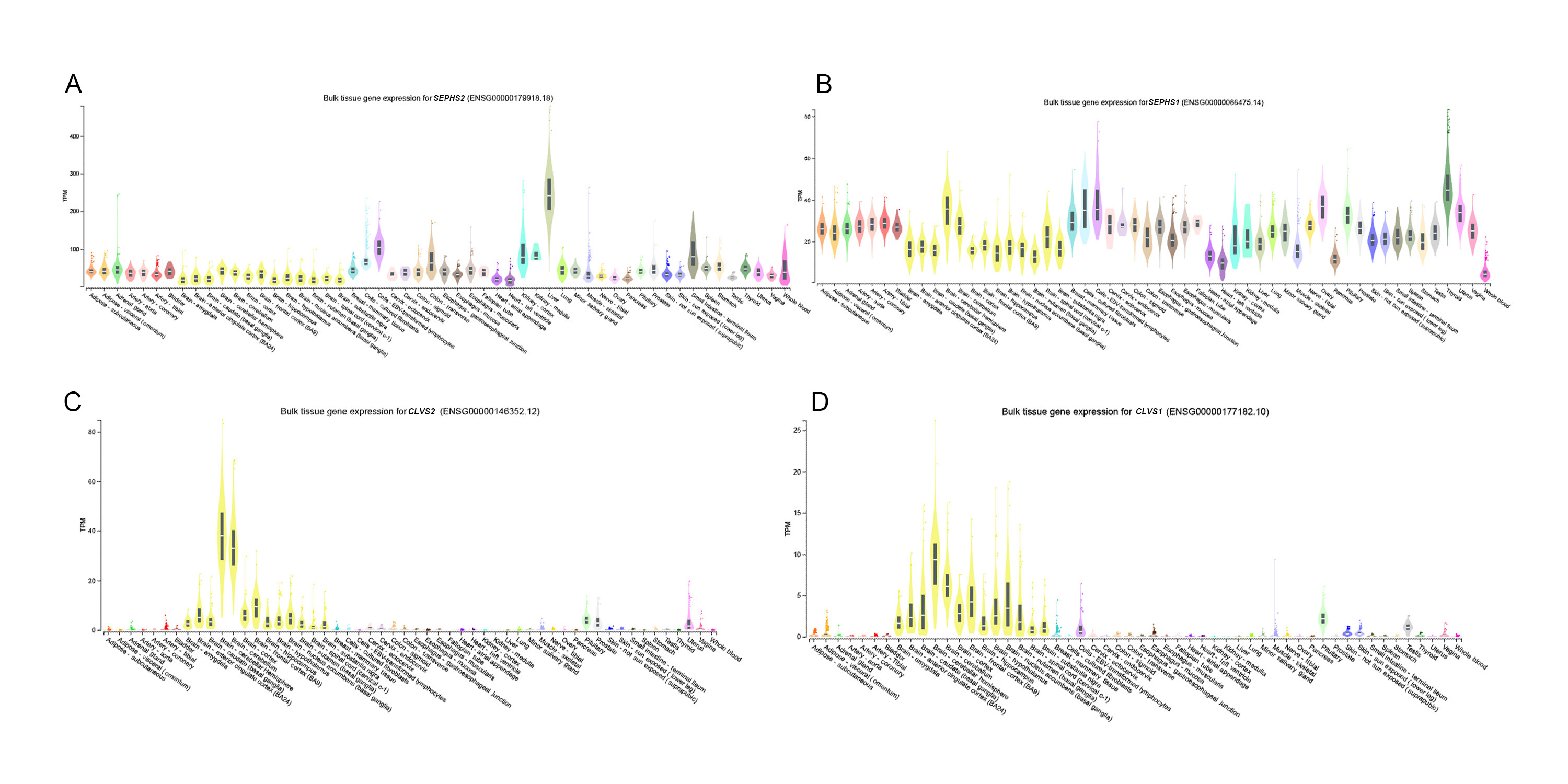

Supplement: qzaf063_Supplementary_Data [file qzaf063_supplementary_data.zip › Supplementary figure 3.jpg]

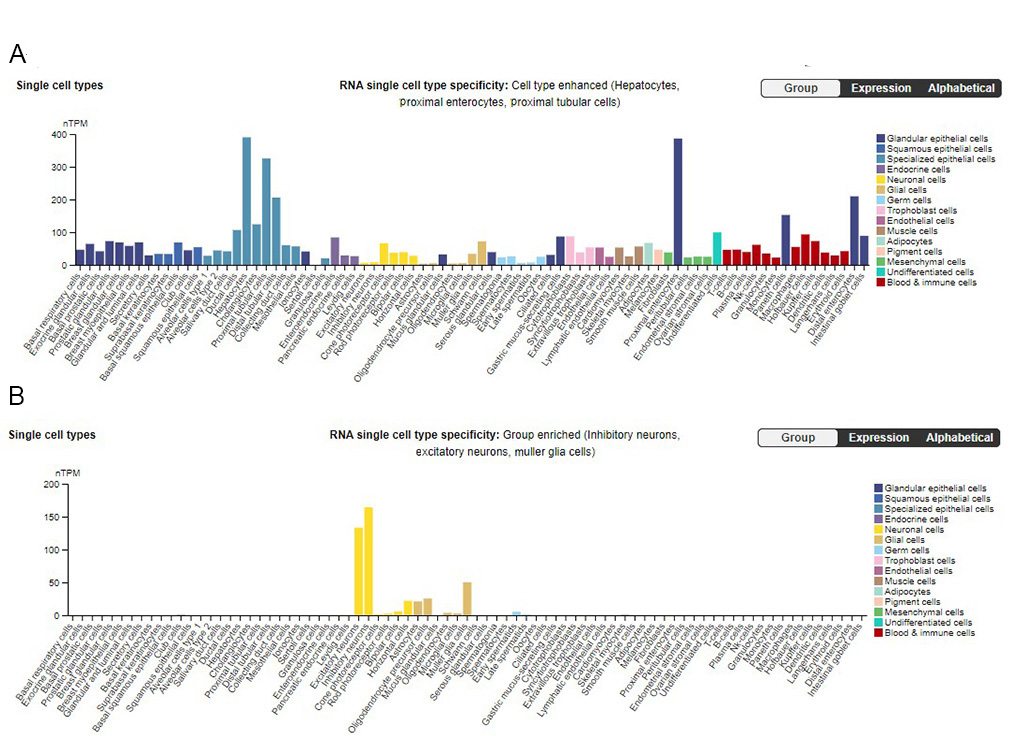

Supplement: qzaf063_Supplementary_Data [file qzaf063_supplementary_data.zip › Supplementary figure 4.jpg]
